# Processing and End-Uses of Wood Affected by Forest Fires, Eastern Spruce Budworm and Mountain Pine Beetle Outbreaks

**DOI:** 10.1007/s40725-026-00273-z

**Published:** 2026-05-22

**Authors:** Rosilei Garcia, Isabelle Duchesne, Louis-David Giasson, Alain Cloutier

**Affiliations:** 1 Present Address: Renewable Materials Research Centre (CRMR), Faculty of Forestry, Geography, and Geomatics, Université Laval, Québec, QC Canada; 2 Canadian Forest Service (CFS), Natural Resources Canada (NRCan), Laurentian Forestry Centre (LFC), Québec, QC Canada; 3 Department of Wood and Forest Sciences, Faculty of Forestry, Geography, and Geomatics, Université Laval, 2425 de la Terrasse Street, Québec, QC G1V 0A6 Canada

**Keywords:** Natural disturbance, Wildfire, Insect outbreaks, Dead trees, Wood quality, Wood products

## Abstract

**Purpose of review:**

This review provides an overview of current forest fires and insect outbreaks in North America, examining how these disturbances affect wood quality over time. It highlights two of the most destructive insect pests: the eastern spruce budworm (SBW) and the mountain pine beetle (MPB). The article also examines the challenges of processing disturbance-affected wood and explores its potential uses in lumber, engineered wood products, and wood-based panels.

**Recent findings:**

Forest fires have intensified and become more frequent in recent years, especially in temperate and boreal forests. Unprecedented SBW outbreaks have also been reported in Eastern Canada. While MPB outbreaks have decreased since 2019, they remain cyclical, indicating the possibility of future resurgences. From 2023 to 2025, these disturbances affected over 60 million hectares in Canada, resulting in significant tree damage and mortality. Although disturbance-affected wood can still be used for lumber and high-value products, its quality deteriorates over time while it remains in the forest, particularly after a fire. Manufacturing products from such wood is difficult due to its low moisture content, changed properties, and high defect rate.

**Summary:**

Disturbance-affected wood typically has lower moisture content, increased permeability, greater brittleness, and a higher incidence of defects, such as insect holes, blue stain, decay, and checks, compared to wood from sound trees. These characteristics can negatively impact lumber recovery and grading and reduce its suitability for secondary manufacturing of wood products like laminated veneer lumber, plywood, or oriented strand board. Nonetheless, this type of wood can be transformed into value-added products such as particleboard, fiberboard, or cross-laminated timber, especially when used in limited proportions or in hybrid compositions. Further research is needed to understand how the properties of disturbance-affected wood change over time and how these changes affect the production of various products. Furthermore, optimizing log sorting, conversion processes, and manufacturing techniques to account for the unique characteristics of disturbance-affected wood is important to ensure the quality and performance of the final products.

## Introduction

Natural disturbances in forests – such as fires, insect outbreaks, diseases, and severe weather events like windstorms and droughts – have intensified and become more frequent in recent decades due to climate change [1]. The latest report from the Food and Agriculture Organization of the United Nations [1] estimates that, on average, 127 million hectares of forests worldwide (based on data from 168 countries, representing 86% of the global forest area) were affected by fires from 2007 to 2019. Furthermore, from 2002 to 2020, an average of 34.1 million hectares of forests per year were affected by insects, and another 16.1 million hectares by disease and severe weather events [1]. These disturbances result in large numbers of damaged and dead trees, leading to substantial economic losses. The disturbances reduce both the quantity and quality of available wood, thereby affecting the annual allowable cut and disrupting the industry’s supply chain. Moreover, such events have significant environmental consequences and pose major challenges for sustainable forest management.

**Fig. 1:**
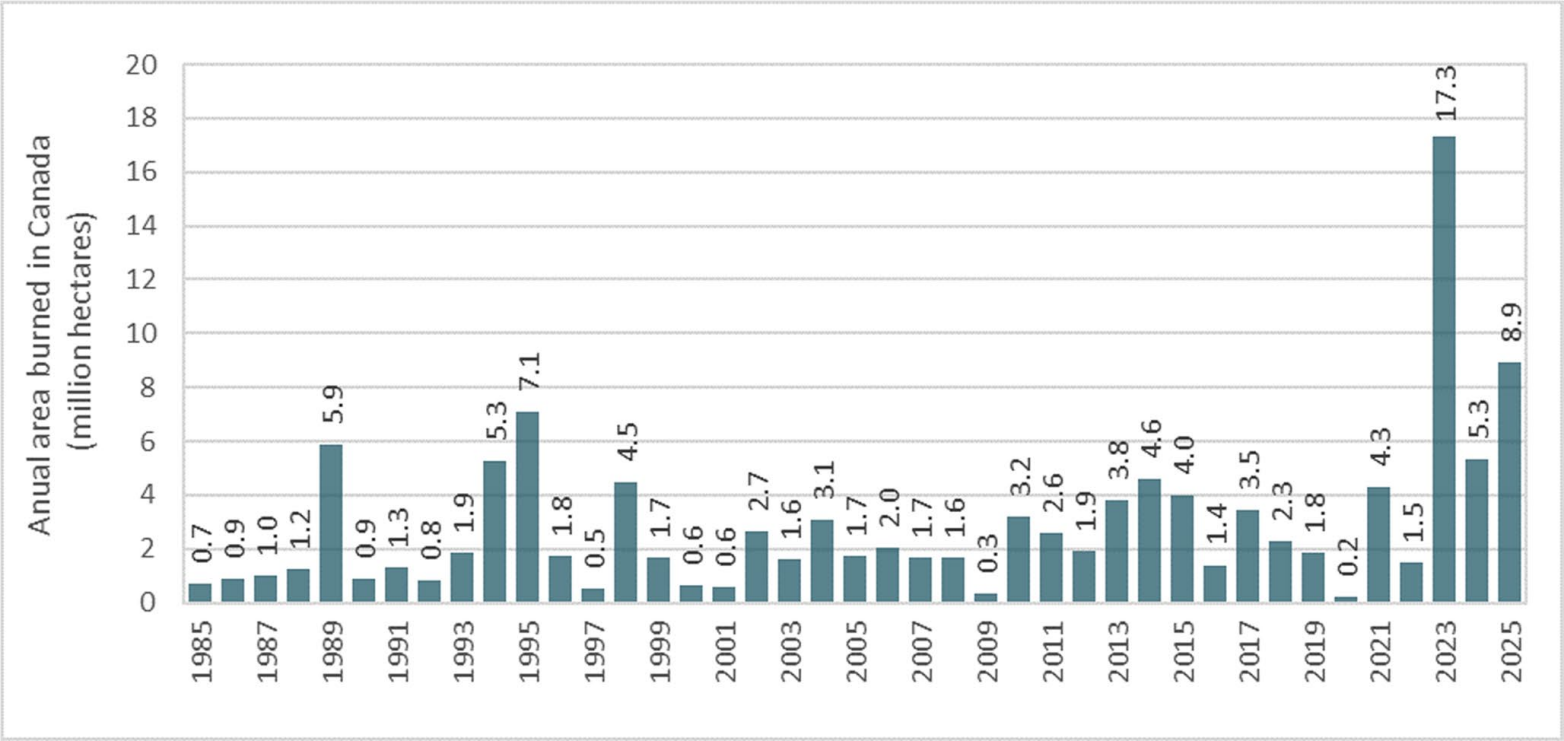
Annual area burned in Canada’s wildlands over the past 40 years. Wildlands encompass forests and other natural ecosystems. Data from the Canadian Interagency Forest Fire Centre [9], as of October 2025

Recent studies report a slight decrease in the global area affected by wildfires, which includes fires in forests, savannas, grasslands, shrublands, and croplands, over the past two decades [2, 3]. However, the intensity and frequency of extreme wildfires have increased significantly in some regions, particularly in the forests of the Northern Hemisphere, resulting in serious environmental, economic, and social impacts [3, 4]. These wildfires have led to considerable biodiversity loss, a significant decline in forest biomass and timber supply, and high carbon gas emissions, in addition to posing threats to human health and safety [2, 3, 5–7]. A recent study analyzing 21 years of satellite data revealed that the frequency and severity of extreme wildfires worldwide have more than doubled between 2003 and 2023, with a significant increase over the last 7 years [4]. The study also showed that wildfire frequency and rate (calculated as the proportion of extreme fires relative to the biome’s area) are worsening in some regions, especially in the temperate coniferous forests of the Western United States and the boreal forests of Alaska, Canada, and Russia. Between 2003 and 2023, the frequency of extreme wildfire events increased by 11.1 times in temperate conifer forests and by 7.3 times in boreal forests [4]. Data from the World Resources Institute [8] shows that Alaska lost an average of 261,000 hectares of forest annually to fire between 2001 and 2024, while California followed with an average loss of 91,900 hectares per year, totaling 8.8 million hectares over that period. Meanwhile, Russia lost approximately 62.3 million hectares of forest to fires between 2001 and 2024, including a record annual loss of 5.36 million hectares in 2021 [8]. In Canada, almost 17 million hectares of forest were burned in 2023, which is 7.9 times the 40-year average. That year set a record for both fire extent and carbon emissions over the past four decades (Fig. 1) [2, 7, 9]. Canada’s carbon emissions in 2023–2024 were over 8 times the average since 2003, accounting for 24% of global emissions during the fire season [2]. The peak fire season in Canada typically starts in early June and lasts for about 14 weeks [8]; however, climate change is causing fires to begin earlier and last longer [2]. By October 2025, nearly 9 million hectares of forest had burned across the country [9]. Jones et al. [2] mention that fire events of a magnitude similar to those that occurred in Canada in 2023 could become 6.3 to 10.8 times more frequent by the century’s end.

These large-scale forest fires have severely disrupted sustainable wood harvesting across Canada, and their cumulative effects are likely to diminish both the availability and quality of wood resources in the long term. In Quebec alone, the 2023 fire events reduced the annual allowable cut by 619,200 m³ for the 2023–2028 period – a 15% reduction of the province’s total – leading to significant socioeconomic impacts in certain regions [10, 11]. They also prompted emergency post-fire logging operations in the northern forest regions, where about 655,000 hectares were affected by the fires [12]. Consequently, post-fire logging has emerged as a practice to mitigate economic losses, maintain the wood supply, and encourage faster reforestation and recovery in certain burned forests. While studies have primarily focused on using fire-damaged wood for pulp and paper, or bioenergy [e.g., 13, 14], fewer have explored its potential for use in lumber and other wood-based products [15–17]. Wood products can store carbon for decades when used in applications such as building materials and furniture, helping to reduce overall greenhouse gas emissions by keeping carbon out of the atmosphere and providing alternatives to materials with high embodied fossil carbon [18, 19]. However, post-fire logging poses complex environmental implications and challenges for the wood industry. On the positive side, it can, for example, help reduce greenhouse gas emissions by preventing the release of CO_2_ and methane – two major contributors to global warming – from burned wood when it is left to decompose in the forest [20]. Burned trees play a vital ecological role in supporting biodiversity, facilitating forest natural regeneration, and contributing to the nutrient cycle through decomposition. They also enrich the soil, promote plant growth, provide food for fungi and bacteria, and serve as a habitat for vertebrates, invertebrates, and birds. Post-fire logging can disrupt these natural processes [21]. It also poses several technical challenges, particularly regarding harvesting time, due to the rapid degradation of the wood. Factors such as fire intensity, fire severity, and the stage of wood degradation influence wood quality and its suitability for various wood products. Despite its importance, this area of research remains relatively underexplored.

**Fig. 2:**
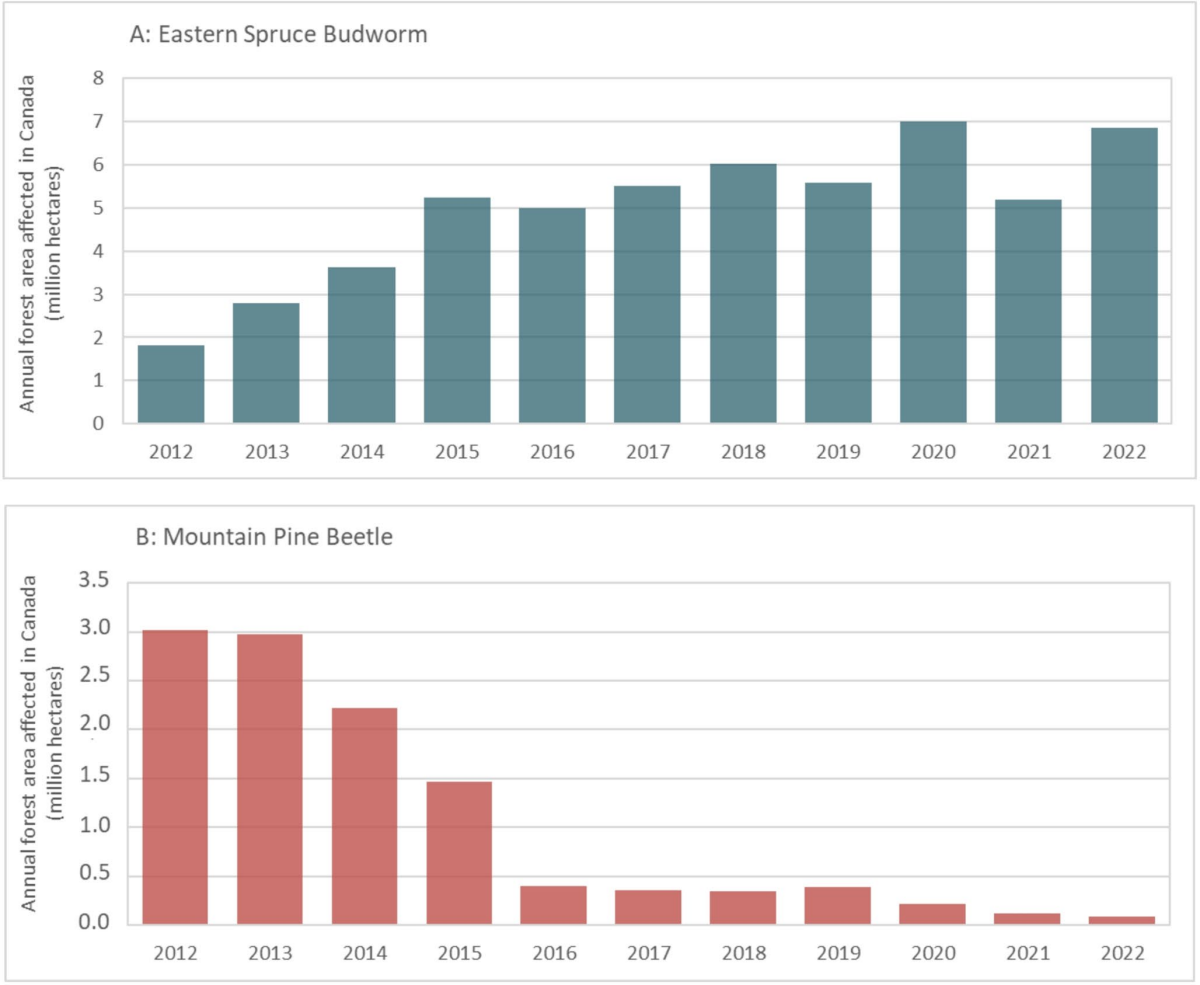
Annual forest area affected by eastern SBW and MPB in Canada between 2012–2022. Data from Natural Resources Canada [22]

Insect outbreaks are the second major cause of natural disturbances in forests. They can significantly impact forests by destroying large stands and reducing the quality of wood. In 2022, Canada reported 13.1 million hectares of forest area impacted by insects, notably defoliators and bark beetles [22]. In North America, the two major insect pests are the eastern spruce budworm – SBW (*Choristoneura fumiferana*; Lepidoptera: Tortricidae) and the mountain pine beetle – MPB (*Dendroctonus ponderosae*; Coleoptera: Curculionidae) [23–25]. An overview of the estimated annual forest area affected by both SBW and MPB in Canada from 2012 to 2022 is shown in Fig. 2. The SBW is a major defoliating insect that stresses trees and reduces tree growth, leading to tree mortality in Eastern North America, particularly in the boreal forests of Canada and the Upper Midwest of the United States, resulting in economic losses and posing operational challenges [26–28]. SBW primarily feeds on the annual foliage of balsam fir (*Abies balsamea*) and white spruce (*Picea glauca*), and to a lesser extent, red spruce (*Picea rubens*) and black spruce (*Picea mariana*) [26, 29]. SBW outbreaks tend to occur in cycles, typically every 30 to 40 years; however, climate change has increased their frequency and severity [24, 27]. For example, in Quebec, a significant increase in the extent and severity of SBW infestations was recorded in 2024. The damage caused by this insect affected 14.3 million hectares in 2024 (ranging from light to severe defoliation), representing a 36.7% increase from 2023 [29].

The MPB is a bark beetle native to Western North America that infests a wide range of pine species, including lodgepole pine (*Pinus contorta*), ponderosa pine (*Pinus ponderosa*), western white pine (*Pinus monticola*), whitebark pine (*Pinus albicaulis*), limber pine (*Pinus flexilis*), and jack pine (*Pinus banksiana*). The MPB does not attack Jeffrey pine (*Pinus jeffreyi*) [25]. These insects live, feed and develop under the bark of trees, where they burrow, lay their eggs, and introduce blue-staining fungi. The larvae that hatch from these eggs consume the tree’s nutrients and moisture, whereas blue-staining fungi disrupt water transport, harming the tree’s growth and leading to tree mortality [25, 30]. In recent decades, massive MPB outbreaks have occurred across the Western United States, Western Canada, and Mexico, primarily affecting mature lodgepole pine stands (> 80 years) [31, 32]. MPB has impacted almost 50% of the area affected by bark beetles in the Western United States [32]. In Canada, the MPB outbreak began in British Columbia in the early 1990 s and subsequently spread to Northern British Columbia and eastward into the boreal forest of north-central Alberta [25]. The last major outbreak of MPBs in British Columbia occurred from 1999 to 2015 [33]. These outbreaks have significantly affected timber supply, impacting approximately 50% of the region’s total commercial lodgepole pine volume. The cumulative loss of pine trees amounted to 752 million m^3^ in 2017, representing 58% of the commercial volume of pine trees [25]. Since 2019, MPB outbreaks have declined in Western Canada (see Fig. 2B); however, these outbreaks are cyclical, occurring every 10 to 30 years [34], suggesting that future periods of resurgence may occur. During MPB outbreaks, the forest industry processed large volumes of damaged and standing dead trees or snags (green, red, and gray stages) to mitigate economic impacts. MPB-affected lodgepole pine is primarily destined for lumber; however, its quality and recovery decline over time, and its processing is challenging. Using MPB-affected wood for engineered wood products and panels, such as oriented strand board (OSB) and medium-density fiberboard (MDF), is technically feasible. However, this approach presents several challenges. The properties of MPB-affected wood differ from those of sound wood, requiring adjustments in both wood processing and product manufacturing [35, 36].

Overall, the increasing amounts of damaged and dead trees from these natural disturbances underscore the need for a better understanding of how these events affect wood quality over time and their implications for the processing and manufacturing of wood products. This knowledge can help identify potential uses for disturbance-affected wood – meaning wood from natural disturbances – at different stages of damage or degradation, enhance process efficiency, and reduce economic and environmental impacts. In this context, this review examines how forest fires and insect outbreaks, specifically SBW and MPB, affect wood quality, and how the timing of harvesting affects it. Additionally, it investigates the potential uses of disturbance-affected wood for various products, including lumber, engineered wood products (cross-laminated timber – CLT, laminated veneer lumber – LVL, and plywood) and wood-based panels (OSB, particleboard, and fiberboard).

## Impact of Forest Fire on Wood Quality

**Fig. 3:**
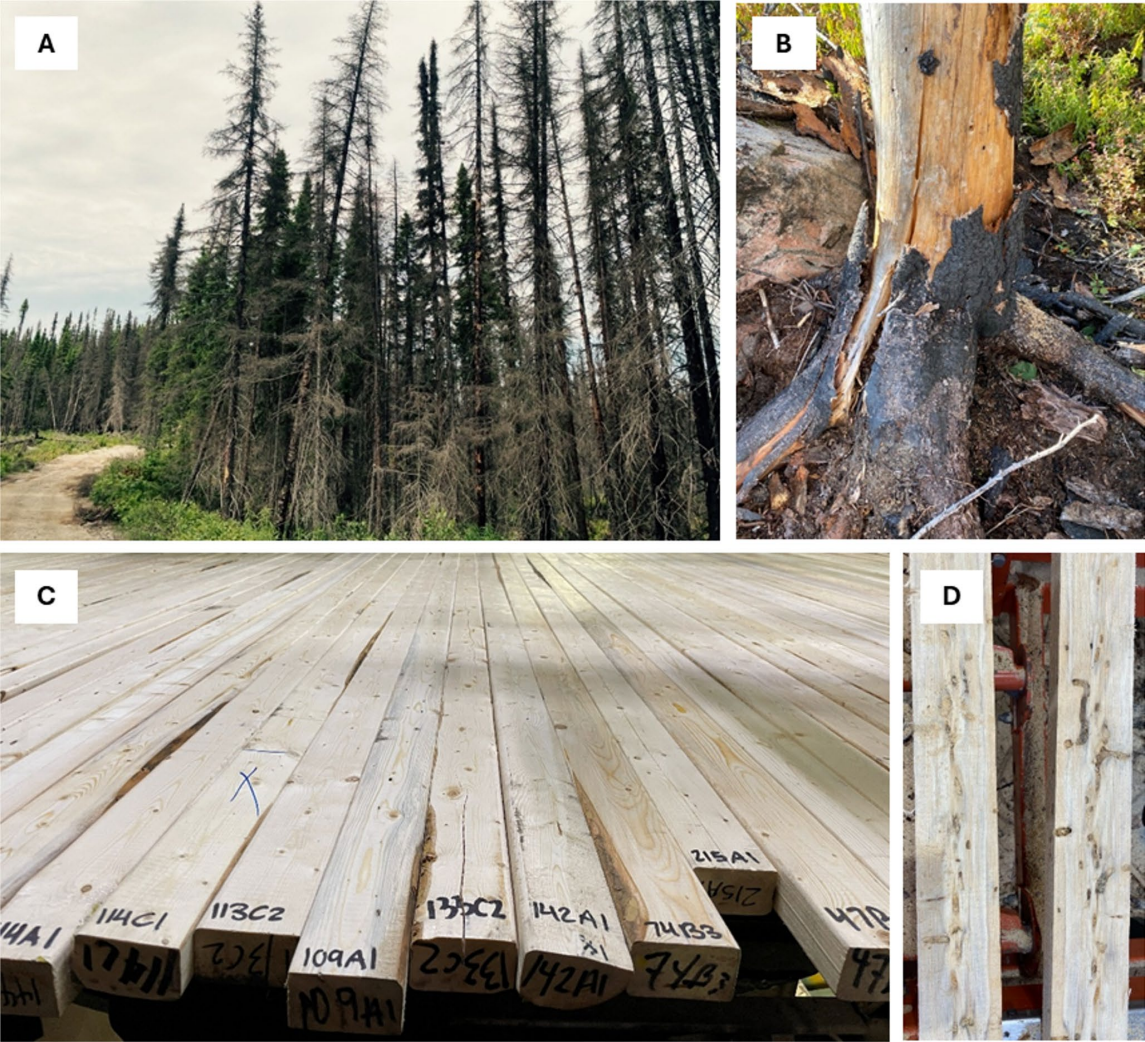
(**A**) A black spruce stand two years after the 2023 forest fires in Chibougamau, Québec, Canada, showing the heterogeneity in tree damage levels. (**B**) A close-up showing bark loss and a deep crack in the trunk of a fire-damaged black spruce tree, two years after the fire. (**C**) The overall appearance of black spruce lumber harvested in the year of the fire. (**D**) Damage caused by the white-spotted sawyer beetle in the heartwood of black spruce, two years after the fire. Photo credit: (A-B, D) Louis-David Giasson; (C) Isabelle Duchesne

The quality of wood affected by forest fires primarily depends on fire intensity and severity, as well as on the extent of wood degradation, which is a time-dependent process. Fire intensity refers to the energy released by a fire and includes elements such as fireline intensity, temperature, and the duration of heat exposure (residence time). In contrast, fire severity measures the extent of damage caused to trees. Although fire severity and intensity are correlated, severity is also influenced by additional factors, such as fuel type, vegetation characteristics, soil conditions, and topography [37]. Therefore, high-intensity fires do not always result in proportionately high severity. Forest fires can be intense and can cover large areas, especially in boreal forests. However, the damage they cause to trees is often heterogeneous, and their effects on wood quality can vary considerably both within a stand and among individual trees (Fig. 3A). This heterogeneity poses a major challenge during harvesting, wood processing, and manufacturing of various products. Fire damage can be assessed at both the stand and tree levels. The Ministry of Natural Resources and Forests of Quebec [29] proposes a burn pattern classification to assess fire damage at the stand level, based on remote sensing, further validated through field sampling, and integrated with forest inventory systems. These burn patterns are classified into four categories: (1) green: unburned, (2) affected: complete or partial spread of fire across the ground with < 50% of the crown scorched or charred, (3) scorched: fire spread over > 50% of the area and with > 50% of the crown scorched, and (4) charred: fire spread over > 50% of the area and > 50% of the crown charred. Classes 2 and 3 are typically associated with low- and medium-to-high-intensity fires, respectively, in which the wood is considered recoverable. Class 4 is associated with high-intensity fires, and the wood is regarded as partially recoverable [38]. At the tree level, fire damage can be categorized using various criteria, including crown conditions (e.g., unscorched, partially scorched, fully scorched, or consumed), bark char height, and other tree features. These criteria provide a baseline for assessing the extent of fire damage to trees, although interpretations can vary across studies.

After a fire, affected trees start to lose moisture. It can take about one year to reach the fiber saturation point (FSP), which corresponds to approximately 25–30% moisture content (MC) based on oven-dry wood weight. However, this duration can vary depending on the tree’s characteristics (e.g., species, age, bark thickness, initial MC, wood density, root depth) and fire severity [39]. Once the MC decreases below the FSP, wood begins to shrink, causing checks and splits [40]. The stress and damage inflicted by fire make trees more susceptible to insect and fungal attack, thereby initiating a degradation process. As this degradation progresses, fire-damaged trees may exhibit various defects, including insect holes caused by wood-feeding beetles (such as woodborers and bark beetles), woodpecker-related damage (foraging holes, nesting cavities, and bark stripping), as well as blue stain, decay, deep checks, and splits. These defects reduce wood quality, affecting lumber recovery, visual and mechanical grades, market value, processing efficiency, and suitability for other wood products, as discussed below.

Insects are typically the first organisms to colonize trees after a forest fire. Woodborers, particularly those from the genera *Arhopalus*, *Melanophila*, and *Oxypteris* (Coleoptera: Cerambycidae and Buprestidae), are quickly attracted to the smoke and heat generated by fires and can reach the affected area within a few hours [41]. Fire-affected trees host more woodborers and have a greater species diversity than trees killed by MPB. Costello et al. [42] found that the woodborer density in fire-affected ponderosa pine trees is three times higher than in MPB-killed trees (13.8 per m² compared to 4.4 per m²). Woodborers can cause structural damage by boring into both the sapwood and heartwood. Several bark beetles (Curculionidae) are also associated with post-fire insect infestations [41]. Although bark beetle damage is often limited to the inner bark (phloem), it can cause tree mortality. Many of these organisms act as vectors that introduce or facilitate the establishment of other pathogens, such as bacteria, fungi, and mites (acari), which are more damaging than beetles. Together, they cause chemical, physical, and mechanical changes in the wood, such as blue stain, drying, and loss of strength properties as degradation advances [43].

Fire-damaged wood is particularly vulnerable to infestations by saproxylic insect species, including the white-spotted sawyer beetle (*Monochamus scutellatus*; Cerambycidae), which is the most damaging wood-boring insect in burned boreal forests of North America [44]. A rapid infestation of these insects was observed in softwoods following forest fires in Eastern Canada during the spring of 2023 [39]. These insects have a two-year life cycle. In the first year, they bore galleries into the outer bark. In the second year, they create deep galleries in the wood, visible as U-shaped in a longitudinal section, which significantly reduces the commercial value of lumber (Fig. 3D) [45]. Various factors, including fire severity, fire season, tree species, and tree characteristics such as bark thickness, diameter, and age, influence the extent of damage caused by the white-spotted sawyer [39, 44, 46]. Their colonization depends on fire severity and tree size [46]. Gervais et al. [46] observed that the number of holes increased in large black spruce trees that were heavily scorched 2 years after a fire. Charred trees, which are associated with high-intensity fires, tend to be too dry for insect and fungal attack, while trees that are slightly affected by fire are less attacked but become more vulnerable after death [39]. Seasonal temperatures also affect the activity of these insects. A study by Bélanger et al. [44] examined the damage caused by these insects at temperatures of 16–28 °C on recently burned black spruce and jack pine logs. The results showed that gallery development occurs more rapidly and at greater depths at temperatures above 24 °C, whereas at 16 °C, larvae were unable to form galleries in the sapwood within the first 200 days. Additionally, black spruce exhibited faster larval development and deeper galleries than jack pine. Their study suggests that severe damage can occur faster after a spring fire, highlighting the need to begin salvage logging promptly [44]. Early harvesting is recommended after spring fire, when white-spotted sawyer attacks may be more severe [39]. For autumn fires, white-spotted sawyer beetle infestation occurs one year later than for spring or early summer fires, allowing a delay in harvest time [13]. Bark thickness is another factor influencing the tree’s resistance to fire intensity and insect infestations. For instance, species with thin bark, such as balsam fir, are more vulnerable to colonization by the white-spotted sawyer beetle after a low-intensity fire, whereas thick-bark species, such as jack pine, require high-intensity fires to facilitate attack by these insects. The damage caused by wood-boring insects, particularly the quantity and size of holes, significantly affects lumber grading [47] by compromising both appearance and mechanical properties. Generally, minimal damage from these insects is observed in the first year after the fire; it is superficial, limited to the inner bark, and can be removed during debarking or log sawing. However, the damage reaches a complete stage 2 years after the fire, resulting in a severe downgrade in lumber quality (Table 1).

**Table 1:** Summary of the effects of forest fire on tree damage and wood quality over time and the subsequent impacts on wood recovery and processing

Time After Fire	Spring Fire	Autumn Fire
Year of Fire (Year 0)	Tree damage: • Woodborer attacks (*Arhopalus*, *Melanophila*,* Oxypteris*) a few hours after a fire. • Bark beetles attack and introduce blue-staining fungi [41]. • Rapid infestation by white-spotted sawyer, which starts to bore galleries in the wood o ↑ colonization with fire severity and tree size [13, 39, 46]. • Possible blue-staining fungi colonization [39]. Impact on wood quality: • ↓ MC, but can stay above FSP • Possible wood discoloration • Lumber: superficial damage, mostly salvageable for quality products [39].	Tree damage: • Similar initial damage: o Woodborer and bark beetle attacks o Possible colonization by blue-staining fungi [41]. • Delayed white-spotted sawyer beetle infestation [39]. Impact on wood quality: • No impact on lumber or wood chips quality [13].
1 Year	Tree damage: • White-spotted sawyer bore deep galleries in wood. • Possible colonization by blue-staining fungi, decay fungi [39]. Impact on wood quality: • ↑ Drying: MC can decrease below FSP → formation of cracks and splits. • Lumber: visual downgrade (staining) o ↓ First-quality lumber (≈ 5% total volume) [39].	Tree damage: • White-spotted sawyer borer starts to create galleries in the wood. Impact on wood quality • Possible wood discoloration • No impact on lumber or wood chip quality [13].
2 Years	Tree damage: • White-spotted sawyer: complete damage • Sapwood decay, wood discoloration • Small-diameter softwood dries below FSP • ↑ Checking and insect colonization [13, 39]. Impact on wood quality: • Lumber: severe downgrade (wood discoloration, cracks, sap rot); first-quality lumber not recoverable • MC > FSP: Use for pulping is possible • MC < FSP: Unusable for pulping [13, 39].	Tree damage: • White-spotted sawyer bore deep galleries in wood • Possible colonization by blue-staining fungi, decay fungi start [39]. Impact on wood quality: • ↑ Drying: MC can decrease below FSP → cracks and splits • Lumber: visual downgrade (staining).
+ 3 Years		Tree damage: • White-spotted sawyer: complete damage • Sapwood decay, wood discoloration • ↑ Brown- and white-rot decay appear vertically, especially in the lower trunk (most valuable part) [39]. Impact on wood quality: • Severe wood damage (checks, decay) [41].
Post-fire logging window	• Short (≈ 1 year) [39].	• Longer (up to 2 years) [39].

Woodpeckers also contribute substantially to the degradation of fire-affected wood. Several species, including the black-backed woodpecker (*Picoides arcticus*), the American three-toed woodpecker (*Picoides dorsalis*), and the hairy woodpecker (*Dryobates villosus*), are strongly associated with recently burned areas, where they forage on wood-boring and bark-beetle larvae. During this activity, they create foraging holes and nesting cavities, causing direct mechanical damage to the wood through excavation and bark removal [21]. This damage further exposes the wood, facilitating fungal colonization and accelerating decay processes [48]. Fire-affected trees in both Western North America coniferous forests and boreal forests are most vulnerable to severe woodpecker foraging during the first one to three years after a fire, when beetle activity and woodpecker occupancy reach their highest levels [49, 50]. Foraging activity is typically greatest within the first three growing seasons following a fire, often resulting in extensive bark scaling, deep excavation holes, and localized sapwood damage [49]. The severity of damage can be influenced by factors such as tree diameter and fire severity [50]. Although woodpecker attack occurs secondary to insect colonization in the wood degradation sequence, their injuries can accelerate drying, checking, sap-stain formation, and fungal ingress, ultimately reducing the recoverable value of sawlogs and veneer.

Blue stain results from ophiostomatoid fungi (Ascomycota and Deuteromycota), which include species from the genera *Ophiostoma*, *Grosmannia*, *Ceratocystiopsis*, *Ceratocystis*, and *Leptographium* [43, 51]. These fungi, well-known for their symbiotic association with bark beetles, colonize wood in the first year after a forest fire [41]. They are called blue-staining fungi because their hyphae spread through sapwood, commonly causing a blue, dark gray, or black discoloration in the wood [43]. Softwood species with larger sapwood bands and large resin canals (e.g., pine species) are more prone to rapid blue-stain fungal growth than species with more heartwood and small (spruce) or no resin canals (fir, genus *Abies*) [52]. Although blue-staining fungi primarily cause aesthetic and economic damage by discoloring lumber, they can also affect other properties of the wood. These include changes to its chemical composition, porosity, permeability, and water absorption (WA), as well as, to a lesser extent, stiffness [51, 53]. Blue-staining fungi do not degrade the structural polymers of wood (cellulose, hemicelluloses, and lignin), but they penetrate through the wood to obtain nutrients, degrading proteins, sugars, starches, resins and waxes stored in parenchyma cells [51, 52]. Fungal colonization also increases wood porosity by degrading pit membranes and forming voids, thereby accelerating moisture uptake in wood [53]. Although the strength properties of wood remain unchanged, a study by Kržišnik et al. [53] showed that blue-stained Norway spruce (*Picea abies*) exhibited a reduction in bending modulus of elasticity (MOE) of 16% compared to sound wood. Furthermore, blue-stained wood exhibited higher surface wettability and WA – twice as fast as sound wood – and lower durability against brown-rot fungi. These characteristics may limit the suitability of blue-stained wood for certain applications requiring durability and moisture resistance, unless appropriate water-repellent and preservative treatments are applied [53]. While the latter research provides important information, no specific studies on blue-stained wood from forest fires were found in the literature.

Decay can be caused by brown-rot or white-rot (Basidiomycota) fungi, which attack different chemical components of wood. Brown-rot fungi, common in North America, mainly colonize softwoods and break down hemicelluloses and cellulose, while leaving lignin mostly intact but with a modified structure due to depolymerization and repolymerization reactions. They cause various types of damage to wood, including shrinking, cracking, brown discoloration due to lignin retention, and reduced mechanical properties [51, 52]. White-rot fungi can decompose hemicelluloses, cellulose, and lignin, enabling complete wood decay. As decay advances, it reduces the strength properties of wood. They occur predominantly in hardwoods but can also attack softwood, mainly sapwood [51]. Decay fungi require specific temperatures, ideally between 5 and 42 °C, and specific humidity and pH levels to attack wood [54]. Brown-rot and white-rot fungi need wood MC above the FSP to grow [52]. The extent of fire damage affects the rate of wood decay. A study by Mușat et al. [55] in a European beech (*Fagus sylvatica*) stand, conducted over 6 years after a litter fire, showed that degradation rates are influenced by the level of bark damage. Trees with exposed wood are more susceptible to xylophagous fungi, resulting in faster decay. In contrast, trees with superficially scorched bark or minimal wood exposure showed only minor impacts on wood quality.

The fire season influences the rate of wood drying, insect and fungal infestations, and decay, as summarized in Table 1. In Eastern Canada, when forest fires occur in the spring, the warm conditions create a favorable environment for rapid insect infestations, particularly by white-spotted sawyer beetles. These conditions also accelerate wood drying, leading to the formation of early cracks and increased decay. Consequently, the window for salvage logging becomes considerably shorter, lasting approximately one year after the fire. In contrast, during autumn, wood degradation tends to be slower, prolonging the drying process, delaying insect activity (e.g., white-spotted sawyer beetles), and hindering decay. These factors can extend the salvage logging window by up to 2 years (Table 1) [21, 39, 44]. While early logging helps prevent wood degradation and minimizes the effects of fire on wood quality, it also poses environmental challenges. Research by Leverkus et al. [56] suggests that delaying post-fire logging for about 2 to 4 years can reduce ecological impacts. Early harvesting may interfere with natural regeneration at certain sites by removing seed-bearing trees, e.g., jack pine and black spruce, which have serotinous and semi-serotinous cones that open in response to intense heat. If specific species, such as jack pine, black spruce, and trembling aspen, can naturally regenerate after a fire, other species like white spruce and balsam fir struggle to establish themselves after fire events. Additionally, post-fire harvesting can impact soil and water quality, stand productivity, and affect the habitat of insects, birds, and other species [21, 41]. It is crucial to recognize that the timing of post-fire logging depends on the specific climatic conditions of each region, meaning these timeframes may not be universally applicable.

## Impact of Insect Outbreak on Wood Quality

### Eastern Spruce Budworm

The SBW does not directly damage the wood, but defoliation can gradually slow tree growth, increase the tree’s vulnerability to secondary insect and fungal attacks, and lead to tree mortality, mainly in mature spruce-fir forests [24]. Few studies have focused on the wood quality from damaged or dead trees following SBW defoliation. Paixao et al. [26] evaluated the effects of SBW defoliation on the growth and wood properties of balsam fir and black spruce. They assessed the effects of a 1- to 4-year defoliation period on ring growth, wood density, and anatomical features. Their results showed that annual radial growth and volume increment decreased with increasing defoliation duration. After 4 years of defoliation, average biomass losses were 34% in black spruce and 31% in balsam fir, based on measurements of annual ring growth over the past 20 years. The growth response to SBW infestation varied among tree species. While the latewood proportion and earlywood density remained unchanged for both species, latewood density decreased after 2 and 3 years of defoliation in black spruce, and average ring density decreased after 4 years. Paixao et al. [26] show that SBW defoliation reduces growth and alters wood anatomy and density, but its direct impact on wood quality appears minimal from a practical standpoint.

**Fig. 4:**
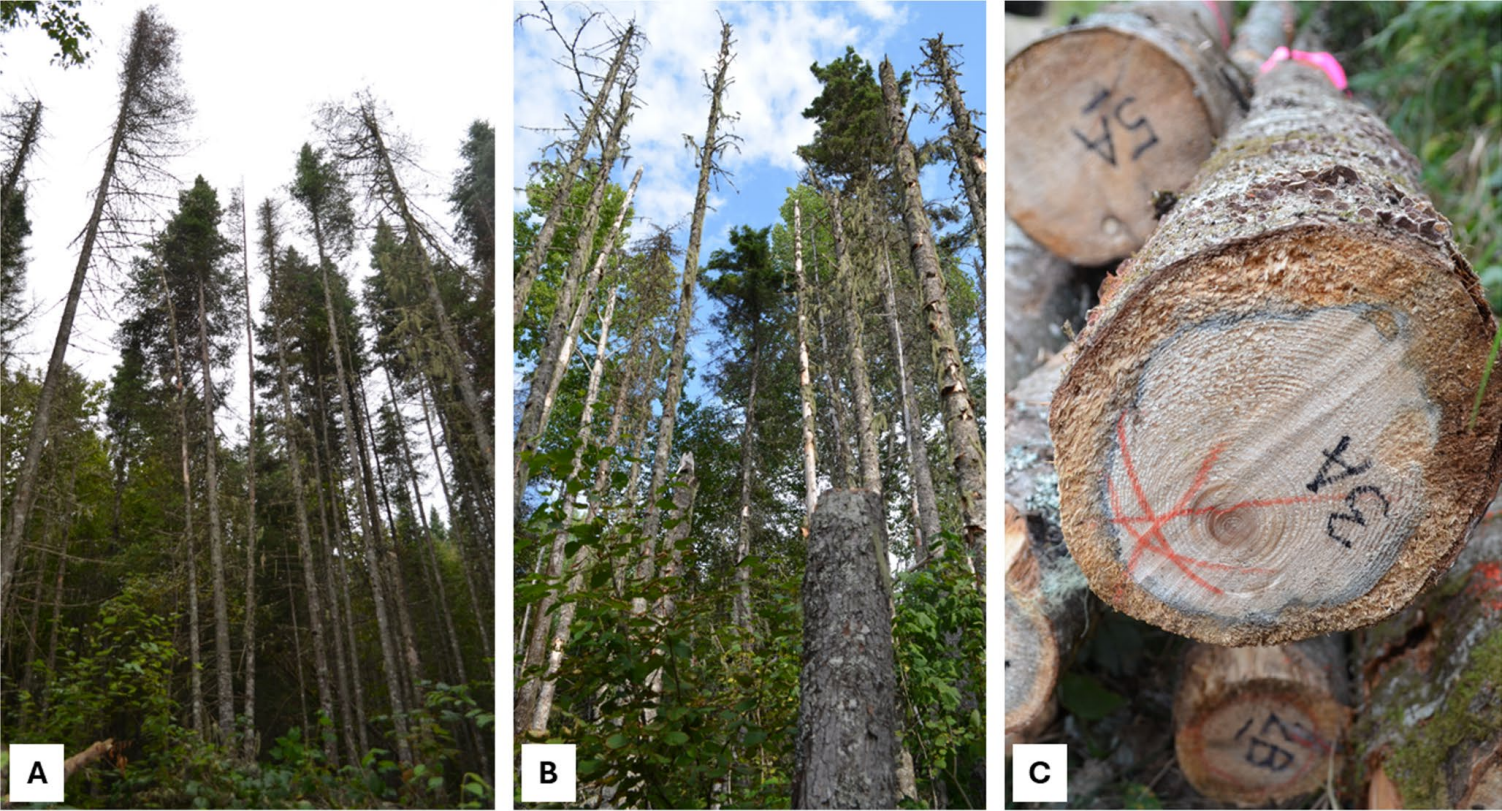
(**A**) Heterogeneous levels of defoliation in balsam fir trees caused by SBW. (**B**) Balsam fir trees after severe defoliation due to SBW. (**C**) Sap rot in balsam fir approximately 6 years after tree death following SBW infestation. Photo credit: Isabelle Duchesne

Trees attacked by SBW typically require several consecutive years of defoliation before mortality; however, the mortality rate varies with factors such as defoliation duration and severity, tree species, age, and forest stand composition [57, 58]. Consequently, there are heterogeneous levels of tree damage within a stand, as shown in Fig. 4A. Generally, mortality occurs after 4 to 5 years of severe defoliation in balsam fir and 6 to 8 years in black spruce [13, 29] (Table 2). Houndode et al. [57] found that mature stands (> 60 years) have a mortality rate that is 5% higher than that of immature stands. Additionally, they found that pure balsam fir stands are more prone to mortality compared to mixed balsam fir-spruce or balsam fir-hardwood stands. After 5 years of defoliation, mortality in balsam fir stands exceeded 22% under severe defoliation and was approximately 12% under moderate defoliation [57]. An exponential increase in balsam fir mortality was observed after 5–10 years of severe defoliation, leading to a decrease in lumber recovery volume [59]. Table 2 summarizes the effects of the SBW defoliation stages (light, moderate, and severe) and the time since death on tree damage, as well as the subsequent effects on wood quality and processing. After death, these trees enter a deterioration phase due to xylophagous insects and fungi, which reduce their suitability for lumber and other applications over time. In the year of the tree’s death, dying trees are invaded by bark beetles, which introduce blue-staining fungi, including *Ophiostoma bicolor*, and various yeast species. This is followed by the invasion of woodwasps (*Sirex* sp.; Hymenoptera: Siricidae) specific to balsam fir, which introduce *Amylostereum chailletti*, a type of white-rot Basidiomycete, and then by sawyer beetles (*Monochamus* sp.). During this period, minimal impact on lumber visual grading and no effect on wood chip quality are observed, since bark beetles and woodwasps damage is limited to the inner bark, and sawyer beetles need a 2-year cycle to bore into the wood [13, 59]. However, blue-staining fungi decrease wood MC, especially after tree mortality, making the wood drier and more brittle. These changes affect various stages of lumber production (e.g., sawing, drying, energy consumption) as well as other manufacturing processes [23, 60]. One year after the tree’s death, sawyer beetle attacks increase, creating U-shaped galleries in the wood and introducing white-rot fungi. At this stage, sapwood discoloration significantly reduces lumber quality, and a lower MC can restrict the use of wood from dead trees for certain processing methods. Two years after the tree’s mortality, colonization by sawyer beetles progresses, and both white-rot and brown-rot fungi spread, causing wood decay at a rate of 10% per year [13, 29, 59]. Sawyer beetles are the primary cause of lumber downgrade 2 years after the tree’s death. After 3 to 4 years, dead trees exhibit high levels of wood decay, and the percentage of standing, intact dead trees decreases over time [59].

**Table 2:** Summary of the effects of SBW defoliation and time since death on tree damage and the subsequent impacts on wood quality and processing

SBW Defoliation Stages		Tree Damage	Effect on Wood Quality and Processing
	Light defoliation	• 1–34% defoliation; loss of annual foliage in the upper third of some tree crowns [38].	• ↓Radial growth and volume increment with duration of defoliation [26]. • After 2–3 years of defoliation: ○ ↓Latewood density (black spruce) [26]. • After 4 years of defoliation: ○ Balsam fir: ↓31% biomass ○ Black spruce: ↓34% biomass, ↓ring density [26]. • Tree death occurs after consecutive years of moderate to severe defoliation: ○ Balsam fir: after 4–5 years; ↑mortality between 5–10 years of defoliation [59]. ○ Black spruce: after 6–8 years [13, 38, 57].
	Moderate defoliation	• 35–69% defoliation; loss of annual foliage in the upper half of the crown of most trees [38].	
	Severe defoliation	• 70–100% defoliation; loss of annual foliage across the entire crown of most trees [38].	
Time after the tree’s death			
	Year of death or dying stage (June–August)	• June: bark beetles invade and introduce blue-staining fungi (*Ophiostoma bicolor*) and yeasts [13]. • July: woodwasps invade balsam fir and introduce white-rot fungi (*Amylostereum chailletti*) [13, 61]. • July-August: sawyer beetles invade [13].	• Significant ↓wood MC from declining to dead trees; MC changes depend on various factors (sapwood-heartwood proportion, tree height, and diameter) [60]. • Sapwood discoloration: ≈ 10–15% of recovery volume [59]. • Impact on wood processing: ○ Minimal impact on visual lumber grading ○ No impact on wood chip quality [13].
	Year 1	• Sawyer beetles create U-shaped galleries and introduce white-rot fungi [7, 13]. • Brown-rot fungi are introduced [13].	• Insect galleries at the base of the trees, sapwood discoloration, and some decay [13, 59]. • Wood MC changes: ○ Balsam-fir: ↓ Sapwood MC: 150–160% (living trees) to 45–80% (dead trees) ↓ Heartwood MC: ≈ 80% (living trees) to ≈ 60% (dead trees) [23]. ○ White spruce: Living trees MC: 77–117% Dead trees: MC varies by height and diameter; large diameter trees (>36 cm) have MC >45%, while small diameter trees (< 17 cm) have MC < 45% [60]. • Mechanical properties: o Dead trees: MOE↓10%, MOR ↓13% [23]. • Impact on wood processing: ○ Significant visual downgrading of lumber [13]. ○ Low MC can restrict the use of wood for certain processes [59].
	Year 2 after death	• Advanced sawyer beetle colonization and white-rot and brown-rot fungi spreading [13, 59].	• Sapwood discoloration and brittleness [13, 59]. • Significant ↓ wood MC: ≈ 45–50% [13, 60]. • ↑Wood decay: ≈10% rate per year [59]. • Impact on wood processing: ○ Greater lumber downgrade: Some large-diameter trees are potentially salvageable for lumber, but at a high loss rate [59]. ○ Low MC: ↑sawing energy, dust issues, more equipment maintenance, ↑sawmill costs [23]. ○ Drier wood: shorter drying times but risk of over-drying → warping and lumber defects [23].
	Year 3–4 after death	• White-rot and brown-rot fungi deterioration progress [13].	• ↑ sapwood/heartwood decay: ≈ 30% • ↓ dead trees remain standing and intact by: ○ ≈ 70% after 3 years of death ○ ≈ 40% after 4 years since tree death [59].

Research has shown that the quality of wood in dead trees following SBW defoliation declines rapidly, mainly due to reduced MC and increased sapwood decay. MC changes in dead trees occur at different rates in sapwood and heartwood and vary with factors such as tree species, height, and diameter [60]. Lemay et al. [23] assessed the wood quality of both living and dead trees in mature balsam fir stands following 3, 4, and 5 years of severe SBW defoliation. The study demonstrated that wood MC was significantly lower in dead trees than in living trees. The sapwood MC dropped from 150 to 160% in living trees to 45–80% in dead trees, regardless of the duration of defoliation. The heartwood MC decreased from about 80% in living trees to 60% in dead trees. Ip et al. [60] also found a significant decrease in MC in dead white spruce trees repeatedly defoliated by SBW. Living trees had wood MCs ranging from 77% to 117%, depending on the proportion of sapwood to heartwood, with no differences in MC between SBW defoliation stages. In contrast, dead trees had much lower MC, which varied with tree height and diameter. For example, dead trees with diameters greater than 36 cm had MC above 45%, whereas dead trees with diameters less than 17 cm had MC below 45%, which is considered too dry (< 45%) for pulping and papermaking processes [60]. As reported by Lemay et al. [23], low MC can also affect log sawing and drying. Logs with low MC generally require more energy for sawing and more maintenance of cutting equipment, which affects sawmill productivity and costs. Drier wood can shorten drying times and reduce drying costs; however, wood from dead trees tends to over-dry, leading to warping and other defects. To minimize lumber defects and processing issues, Lemay et al. [23] recommend harvesting as soon as possible after the tree’s death. In terms of mechanical properties, decreases of 10% and 13% in bending MOE and modulus of rupture (MOR) were observed in dead trees. However, no significant differences were found between defoliation periods [23]. Overall, low MC and the tendency of decayed wood to produce fine particles are crucial factors to consider in wood processing and the manufacturing of wood products from trees affected by SBW. Wood volume losses during logging, transportation and debarking are also among the main constraints associated with harvesting dead trees from SBW infestations [59].

### Mountain Pine Beetle

The quality of wood following MPB attack declines over time due to blue stain, drying, decay, and checking, which can reduce its suitability for lumber and other wood products [31]. Trees affected by MPB progress through three visual stages, from living to dead, which are classified as follows: green stage – corresponding to the first year of MPB attack, during which trees have green and moist needles; red stage – over the next 2 to 3 years, the needles dry out and turn red before falling to the ground; and gray stage – in which trees lose all their needles. Trees can remain in the gray stage for many years before falling to the ground (fall stage). Most wood properties change from the green to the gray stage, particularly during the first 2 years after the tree’s death [62]. A significant increase in blue-staining fungi and a decrease in wood MC occurs in the first year (green stage), followed by a notable increase in checks from the second year after MPB attack (red stage), and progressive decay from the red to the gray stage [63]. Table 3 summarizes the damage caused to trees by MPB and the subsequent effects on wood quality and processing during green, red, and gray stages.

**Table 3:** Summary of the effects of MPB outbreaks in pine species at green, red, and gray stages and the subsequent impacts on wood quality and processing

Tree Visual Stages		Tree Damage	Effect on Wood Quality and Processing
	Green (First year following MPB attack)	• Rapid colonization by blue-staining fungi, mainly *Ophiostoma clavigerum* and *Ophiostoma montium* (known as deep stainers), disrupts water transport and ↓MC in sapwood [30]. • Presence of a small number of secondary beetles (Ips, ambrosia beetles) [30, 42, 68]. • Presence of *Entomorticium* species, very slow-growing decay fungi, not causing significant fiber degradation [68]. • Decay fungi are rare [30].	• 2–6 weeks post-attack: blue stain (sapwood) [30]. • ↓ sapwood MC from 120–130% to 40-80% within 1 year [30]. • Eight months post-attack: ○ Sapwood, heartwood: significant ↓MC, ↓specific gravity ○ Sapwood: ↓extractives, carbohydrates, and lignin, ↑ longitudinal permeability [64]. • No signs of checking, sap rot, or woodborer damage (measured in basal discs) [62]. • Logs retain most of their commercial value [67].
	Red (Year 2–3 following MPB attack)	• ↓blue-staining fungi frequency, secondary fungi appear (*Leptographium terebrantis*, *Ophiostoma minus*, *Ambrosiella* sp) • ↑ prevalence of decay fungi (*Fomitopsis pinicola*, *Heterobasidion annosum*) • ↑ fungal diversity and number of secondary beetles [30].	• Significant ↓ wood MC: ○ Sapwood MC: near to FSP (≈25%) ○ Heartwood MC: ≈ 22–36% [62, 68]. • ↑ percentage of trees with sap rot and woodborer attacks [62]. • Checking: >70% of trees develop checks in mid-stem in year 2 [62]. • Impact on processing: ○ Recoverable for lumber, but ↓ visual grading due to blue stain [63]. ○ ↓15% sawlog volume compared to green trees [67].
	Gray (Year 4 or more following MPB attack)	• Significant ↓ in wood MC; MC too low for some fungal colonization [30]. • Absence of decay fungi colonization in red and grey trees with MC < 20% [68].	• Sapwood MC: few differences between red and grey trees [68]. • Heartwood MC: ≈ 15–36% [68]. • Severe deterioration; ↑ decay over time after tree death [67]. • Impact on processing: ○ ↓49% sawlog volume compared to red trees [67]. • After 5–7 years: ↑ tree falling, resulting ↑ in safety risks for salvage logging [63].

MPBs are associated with several ophiostomatoid and basidiomycetous fungi. Following MPB attack, lodgepole pine can be colonized by blue-staining fungi within a few weeks (approximately 2–6 weeks). During the early green stage, two deep-staining fungi (*Ophiostoma clavigerum* and *Ophiostoma montium*) rapidly colonize trees, causing sapwood discoloration [30]. MPB and blue-staining fungi have a mutualistic association, in which these fungi help decrease wood MC and create favourable conditions for beetle reproduction. The damage caused by blue-staining fungi to trees is more severe than that caused by MPBs, as they disrupt water transport and can lead to tree death. Consequently, wood undergoes a significant decrease in MC, a major challenge for sawmills when processing logs from red- or gray-stage trees [30].

Trees infested by MPB exhibit a gradual decline in MC as they progress from green to gray stages, with a more significant decrease in sapwood compared to heartwood. Sapwood MC decreased from over 43% in green-stage trees to about 22% in red-stage and 15% in grey-stage trees. Meanwhile, heartwood MC decreased from approximately 34% in green-stage trees to 25% in red-stage and to 17% in gray-stage [30]. Woo et al. [64] investigated the chemical and physical properties of mature lodgepole pine trees aged 85–95 years, harvested approximately 8 months after an MPB attack. They observed a significant decrease in the MC of both sapwood and heartwood in affected trees with increasing tree height. Compared to sound wood, the MPB-affected lodgepole pine exhibited lower specific gravity in sapwood and heartwood, attributed to fungal degradation, and the sapwood had lower extractive, lignin, and carbohydrate contents. Furthermore, blue-stained sapwood exhibits increased permeability and diffusion coefficients, which can influence the lumber-drying process and chemical treatments [65, 66]. According to Cai and Oliveira [66], blue-stained lodgepole pine exhibits radial permeability 2.4 to 23 times higher than non-stained wood, and its tangential permeability is 5 to 13 times higher compared to non-stained wood. Also, the diffusion coefficients in blue-stained wood were 23.5–70.5% higher within temperatures ranging from 50 to 90 °C, which impacts preservative treatments. Woo et al. [64] also observed different longitudinal permeability patterns in MPB-affected wood, with higher permeability in sapwood due to fungal degradation of pit membranes and ray parenchyma cells, and lower permeability in heartwood compared to sound lodgepole pine. Longitudinal permeability was also found to vary with tree height and to be negatively related to extractive content.

The percentage of trees exhibiting checking, sap rot, and woodborer damage increased over time since the tree’s death at nearly the same rate as moisture loss [62]. In the study by Lewis and Thompson [62], checks developed rapidly within the first 2 years following tree death. A positive correlation was found between the number and depth of checks, the depth of sap rot, and the extent of woodborer damage, related to the time since death and the tree’s diameter. These findings indicate that large trees must be harvested within 2 years of MPB infestation, whereas delayed harvest is possible for small-diameter trees (6 years or more after MPB attack) [62].

A wide diversity of decay fungi has been found in lodgepole pine trees attacked by MPB; however, this diversity varies with site, tree stage (green, red, or grey), density, and MC [68]. Breuil [68] identified 40 basidiomycete species across 12 sites in British Columbia in trees that were affected by MPB. The most commonly isolated fungi from both living and dead trees across all sites were *Entomocorticium* species. These very slow-growing decay fungi do not cause significant fiber degradation and are rather a source of nutrients for beetles. In contrast, an increased prevalence of aggressive species such as *Fomitopsis pinicola* (brown-rot fungi), *Ganoderma* sp., and *Melutlodontia* sp. was identified in the red stage [68]. In the gray stage, severe wood deterioration is observed, reducing the volume of recoverable wood by up to 49% [67]. However, the decay rates are influenced by various factors, such as wood MC, wood density, tree size, climate and site conditions. Some species of decay fungi can cause wood weight loss of up to 50–60% within 12 weeks at 20 °C [68].

## Comparison of Forest Fires, Eastern Spruce Budworm, and Mountain Pine Beetle Impacts on Wood Quality

Table 4 provides a comparative overview of the impact of forest fires and SBW and MPB infestations on wood quality. While these disturbances can cause similar wood defects, such as insect holes, wood discoloration, checks, decay, and reduced strength, they differ in their mechanisms of damage and the timing of wood degradation. The impact of forest fires is immediate, leading to the rapid deterioration of wood quality. In contrast, the effects of SBW and MPB infestations are characterized by gradual deterioration, with MPB causing tree death more rapidly than SBW.

**Table 4.**
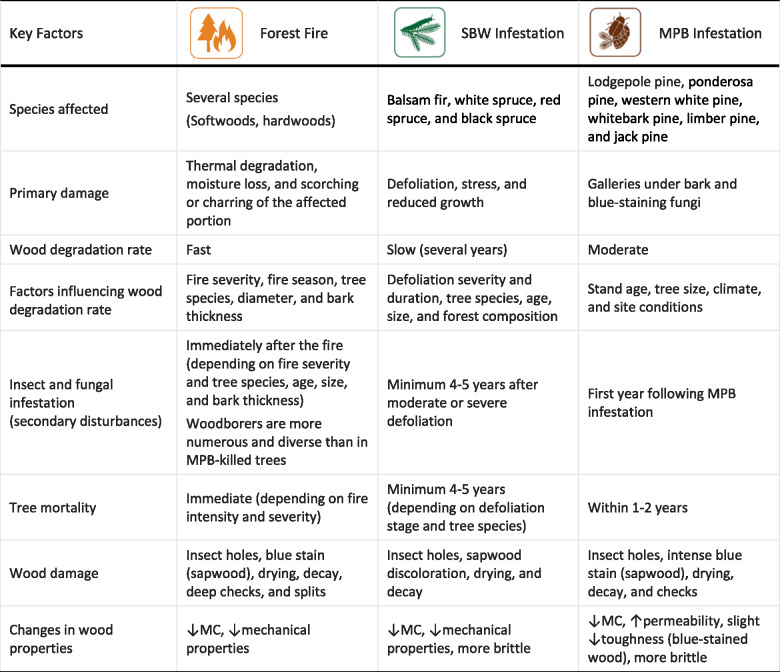
Comparison of forest fire and SBW and MPB infestations' impacts on wood quality [25, 26, 29, 39, 42, 63, 68–73].

## Wood Products from Damaged and Dead Trees

Research on the use of damaged or dead trees following forest fires and SBW or MPB infestations in wood products is limited. Among the 24 publications summarized in Table 5, only six have been published in the past five years [see 15, 27, 70, 73, 98, 99]. Among these six publications, three specifically focus on forest fires (lumber), while one addresses SBW infestation (lumber, CLT). The remaining two publications examine dead trees without determining the cause of their mortality and focus on a different species of bark beetle. Out of the other 18 publications, 11 focus on MPB, most published during the 1999–2015 MPB outbreak. While these publications may not be recent, they still provide valuable insights applicable to other disturbance sources and can guide future research.

**Table 5:** Summary of the effects of using damaged or dead trees on wood processing and product properties.

Wood Product	Disturbance	Species Affected | Harvest Timing	Focus of the Study	Impact on Wood Processing and Product Properties	Reference
Lumber		• Norway spruce, Scots pine • Harvested 4 months after a high-intensity fire	• Suitability of fire-damaged wood for sawn timber • Physical and mechanical properties	• Darker color, ↓MC (spruce), slight ↑Brinell hardness (spruce), minor ↓MOR (pine) • Suitable for medium- to lower-grade products • Impact on wood processing: ○ Char and soot contamination ○ Requiring technology to reduce emissions	[15]
		• Caribbean pine • Harvest time not specified	• Mechanical properties	• ↓10% Density, ↓15% MOE, ↓13% compression strength parallel to the grain • Limitations for structural uses	[70]
		• Loblolly pine • Harvested 1 month after the fire	• Mechanical properties, chemical composition • 4 levels of crown damage	• No impact on specific gravity, shrinkage, compression strength perpendicular to the grain, shear strength • Severe fire damage: o ↓Bending properties, compression strength parallel to the grain, toughness o ↓Cold-water extractive content • Limitations for structural uses	[71]
		• Maritime pine • Harvested 5–7 months after a high-intensity fire	• Mechanical properties	• ↓Bending properties, compression strength, and toughness due to post-fire degradation, not the direct effect of heat	[72]
		• Scots pine • Medium-intensity fire • Harvest time not specified	• Mechanical properties • Bottom, middle, and top parts of the trunk	• Thermal degradation: ↓MC, ↑brittleness • ↓Bending properties, compression strength parallel to the grain, tension strength parallel to the grain • Strength changes: more pronounced in the top part of the trunk than in the bottom	[73]
		• Three oak species • Harvested 1–14 years after the fire	• Effect of fire-scar size and post-fire harvest timing on lumber volume and value	• Lumber volume: minimal losses with fire-scar height ≤50 cm and basal circumference loss ≤ 20% (up to 14 years after fire) • ↓Lumber value over time o Early harvest (≤ 5 years): slight ↓ lumber value in severely damaged trees, regardless of scar size	[76]
		• White spruce • Harvest time not specified	• Dynamic MOE of living and dead trees following SBW/beetle attack	• MOE: ↓6.6% (recently dead) and 4.5% (long dead) compared with living trees, with no difference between dead-tree categories	[27]
		• Black spruce • Cause of the tree’s death not specified • Harvest time not specified | Based on Hunter classes	• Bending properties • Living trees vs. dead trees (Hunter 3) and dead trees with bark peeling (Hunter 4)	• ↓MOE/MOR in 2x4 and 2x6 lumber from dead trees (Hunter 3 and 4) compared with lumber from living trees	[77]
		• Black spruce • Cause of the tree’s death not specified • Harvest time not specified | Based on Hunter classes	• Lumber grade yield and value • Living trees vs. recently dead trees (Hunter 3) and dead trees with bark peeling (Hunter 4)	• ↓Lumber quality: ≈ 74% lumber graded as No.2 and better (live trees) vs ≈ 60% (Hunter 3) and ≈ 40% (Hunter 4) • Hunter 3: profitable, Hunter 4: poor lumber quality, low returns; should remain in the forest for ecological benefits • ↑Decay and breakage with Hunter class	[78]
		• Lodgepole pine • Harvested at green, red, and gray stages	• Wood processing issues, processing costs, product value	• Impact on wood processing: o Issues: breakage during handling and sawing, dust problems, sorting complexity o Equipment wear and kiln-drying time are minimally affected • ↑Cost: ≈ 15% (red stage), ≈ 30% (gray stage) compared to the green stage • Product value: cracks/checks are the most damaging defects; blue stain has minimal impact	[67]
		• Lodgepole pine • Harvest time not specified	• Blue stain impact on mechanical properties	• Blue stains: minimal impact on wood strength with slight ↓toughness, slight ↑MOE, better connector grip • Mechanical properties differences are often masked by the natural variability of wood • Acceptable for structural use under standard grading rules	[80]
		• Lodgepole pine • Harvest time not specified	• Humidification systems for heat treatments to meet phytosanitary regulations	• MPB lumber issues: too dry, standard kiln drying/heat treatment schedules are long, resulting in over-drying and ↓ lumber quality • Low-pressure steam or cold-water spray: ↓kiln residence time, minimize or eliminate over-drying, ↑lumber quality	[81]
		• Lodgepole pine • Harvest time not specified	• Heat treatments • Mechanical properties and appearance (blue stains)	• Darkened wood: reduce or eliminate blue stain • Improved dimensional stability: ↓volumetric swelling • ↓MOE/MOR	[85]
CLT		• White spruce • Harvest time not specified	• Bending and shear properties of living and dead trees following SBW/beetle attack	• Slight ↓ bending properties in dead-tree categories, but still compliant • All panels exceeded Grade E3 for MOE and met Grade E1 for MOR according to the ANSI/APA PGR 320-2019 standard • Shear properties: ↓ ≈ 22% (recently dead), ↓ ≈ 17% (long-dead)	[27]
LVL		• Lodgepole pine • Harvest time not specified	• Veneer PF impregnation • 2 veneer grades: E1 (high grade), E2 (low grade) • LVL layup optimization	• E2: ↑resin absorption due to ↓veneer density, ↑permeability • PF-impregnation: ↓TS/ WA, ↑surface hardness of LVL • ↑ MOE: one-layer impregnated (E1), or double-layer impregnated (E2) on face and back sides	[94]
		• Lodgepole pine • Harvest time not specified	• PF impregnation, veneer properties, LVL performance	• PF resin masked sapwood stain, ↑40% MOE for the best group, ↑surface hardness (up to 46%), ↓TS/WA • More resin uptake in stained compared to non-stained veneers	[95]
Plywood		• Lodgepole pine • Harvested within 1-2 years after MPB attack (green and red stages)	• Logs peeling: MPB vs S-P-F logs • Veneer sorting and processing	• MPB logs: ↓MC but less MC variation than S-P-F logs, which improves veneer sorting efficiency; cracks and dryness cause veneer ribbon breakage during peeling; blue stain comprised ≈ 50% total log volume; veneer recovery ↓ ≈ 8% than S-P-F logs • MPB veneer: ↓25-35% drying time, shrinks less in width, but ↓visual grading (blue stain)	[96]
		• Lodgepole pine • Harvest at green and red stages	• Plywood manufacturing, veneer grading, gluing, panel lay-up, hot pressing • MPB plywood vs S-P-F plywood	• MPB veneers: exhibit blue stain, cracks, roughness, but no strength impact • MPB plywood: ↑glueability, ↑≈ 14-20% MOE/MOR compared to S-P-F plywood • Manufacturing: requires ≈ 10% longer pressing time • Lay-up: stained veneers should be placed in inner layers; need for improvements in vision systems for sorting with color saturation algorithms	[93]
		• Lodgepole pine • Harvest time not specified	• MPB-affected lodgepole pine vs Douglas-fir veneers • Manufacture of specialty plywood	• MPB plywood: better glueability but ↑TS/WA compared to Douglas-fir • Mixed composition: Douglas-fir (outer layers), MPB veneer (inner layers): enhanced appearance, dimensional stability, and overall performance • MPB veneers: acceptable for inner layers of specialty plywood	[97]
OSB		• Jack pine (charred bark removed) • Harvested 3 weeks after the fire	• Physical, mechanical, chemical properties • Panels bonded with PF • 3 burnt patterns	• No impact on MOE, MOR, IB, regardless of the burnt pattern • All panels met the CSA O437 mechanical standard • Severely damaged trees: ↑Klason lignin, ↓glucose, improved water resistance, and a tendency to ↓MOR/MOE	[16]
		• Red pine • Harvested within 2 months after the fire	• Physical, mechanical, chemical properties • Panels bonded with pMDI • Fire severity, bark content	• Up to 10% bark: no negative effect; > 10% bark: ↓MOR/MOE • MOR, MOE, IB met CSA requirements, regardless of fire-damage level • Bark: ↓TS/WA, but most panels exceed CSA TS limits	[16]
		• Lodgepole pine • Standing dead trees for 20 years (gray stage)	• Improve the strands quality • Log rehydration treatments	• 10 days of water spraying: achieved MC in sapwood like freshly cut logs, ↓28% fines, ↑45% large strands • Strand quality can be improved when heartwood MC approaches FSP	[31]
		• Lodgepole pine • Harvest time not specified	• Dimensional stability • Different coatings applied to industrial panels	• Some commercial waxes and linseed oil-based combined with carnauba, paraffin, or expandable microspheres provided short-term water resistance (up to 24 h) • Performance declines after long-term water exposure	[36]
Particleboard		• Mixed softwood-hardwood • Cause of the tree’s death not specified • Harvest time not specified	• Physical and mechanical properties, formaldehyde emissions • Various proportions of dead-tree wood particles	• Up to 25% dead-tree wood particles: no property impact • Panels made from 100% dead-tree particles: ○ ↓27% MOR, ↓23% MOE, ↓74% IB, ↑21% TS, ↑11% WA ○ 3% MUF: ↑properties ○ Formaldehyde emissions: ↓35% (UF), ↓39% (UF+MUF)	[98]
MDF		• Scots pine • Harvest time not specified	• Physical, mechanical properties • Surface properties, aesthetics • Various burned fiber proportions (0-100%)	• ↑77-143% TS, depending on burned fiber proportion • Unchanged MOR, MOE, IB; ↑screw holding capacity (> 75% burned fiber); ↓Janka hardness (100% burned fibers) • ↑Surface roughness, darker coloration • Up to 50% burned fibers can be used in MDF production.	[17]
Fiberboard (Wet process)	Bark beetle *Polygraphus proximus*	• Siberian fir • Various trees with dead periods up to 19 years	• Refining (quality, power consumption), fiber size • Physical and mechanical properties	• Long-dead trees: ↑fiber refining, ↓70% energy consumption • Dead trees (5–12 years): high-quality fibers, ↑panel strength • Dead trees (19 years): ↑proportion of small fibers, ↓TS, surface defects (dark spots) • ↓Specific energy consumption, ↑refining efficiency with time since the tree’s death.	[99]

### Lumber

One of the few studies on the suitability of fire-damaged wood for lumber was published by Martilla et al. [15]. They evaluated the physical and mechanical properties of Norway spruce and Scots pine (*Pinus sylvestris*) harvested 4 months after an intense forest fire in Western Finland, focusing on bending properties, Brinell hardness, MC, and color. Both species had a darker color, but the MC decreased only in spruce. The mechanical properties remained almost unchanged, with minor increases in Brinell hardness for spruce and decreases in the MOR for pine. The study also highlighted the effects of bark thickness and crown shape on the extent of fire-induced wood damage. Norway spruce, which has thinner bark and a longer crown, exhibited more severe fire damage than Scots pine [15]. These findings are consistent with earlier research [74], which found that species with thin bark, such as spruce and birch, are particularly vulnerable to fire. Kolström and Kellomäki [74] also found that fire tolerance increases with tree size, highlighting the importance of considering species- and age-related factors when evaluating the quality of fire-damaged wood. The main challenge in processing fire-damaged wood, particularly spruce, was contamination with char and soot, which affected both the lumber and sawmill equipment [15]. Dust contamination not only affects the quality of the final product but also increases the demand for equipment maintenance, requires frequent cleaning breaks, and can pose respiratory issues for workers [15, 75]. Additional challenges include debarking efficiency, as charred bark can hinder mechanical removal. Moreover, variable MC, which changes with fire intensity and severity and time since the fire, complicates kiln drying and may result in under- or over-drying of lumber [75]. These issues underline the need for equipment that minimizes or eliminates char and soot emissions while effectively processing fire-damaged wood in sawmills [15, 75].

Other studies have shown that fire significantly reduces certain mechanical properties of pine wood [70–73]. Zziwa et al. [70] evaluated the mechanical properties of 10-year-old Caribbean pine trees affected by a forest fire and found significant reductions in density, bending MOE, and compression strength parallel to the grain, with decreases of 10%, 15%, and 13%, respectively. Another study by Bortoletto Júnior and Moreschi [71] assessed the mechanical properties and chemical composition of 23-year-old loblolly pine wood one month after a forest fire. The trees were free from fungal or insect infestations. Fire-damaged wood was categorized into four fire severity levels based on crown damage: (1) superficial burning without lethal crown scorch, (2) superficial burning with partial lethal crown scorch; (3) partial crown consumption; and (4) total crown consumption. Across all damage levels, no significant differences were found in specific gravity, shrinkage, compression strength perpendicular to the grain, or shear strength. However, the bending MOR/MOE, bending toughness, and compression strength parallel to the grain were reduced in fire-damaged wood from level 4, which can limit its use in structural applications. Regarding chemical composition, only a decrease in cold-water-soluble extractive content was observed in the most severely damaged wood. The authors attributed the relative preservation of wood properties to the protective role of the thick bark, which limits heat penetration. Carvalho [72] also reported that maritime pine (*Pinus pinaster*) harvested 5 to 7 months after a high-intensity forest fire showed reduced bending properties, parallel-to-grain compression, and toughness, mainly due to post-fire degradation rather than direct fire damage. Similarly, Lukina et al. [73] found significant decreases in the mechanical properties of fire-damaged Scots pine wood after a medium-intensity fire.

Marschall et al. [76] evaluated how fire-scar damage affects the volume and value of lumber for three oak species – black oak (*Quercus velutina*), northern red oak (*Quercus rubra*), and scarlet oak (*Quercus coccinea*). They developed models to predict how the size of fire scars and the timing of post-fire harvest influence the wood’s value. The study evaluated trees with a minimum diameter of 20 cm harvested from three sites in southern Missouri, following fire events that occurred 1–14 years earlier. Losses in lumber volume and value were minimal for trees with fire-scars located below 50 cm or with up to 20% loss in basal circumference during the 14 years following the fire. Beyond these thresholds, lumber value tended to decrease over time. When harvesting occurred within 5 years, wood value decreased slightly regardless of scar size.

With respect to lumber from insect outbreaks, Ma et al. [27] evaluated the mechanical properties of lumber from dead white spruce following an SBW infestation. They compared the dynamic MOE of lumber from living trees, recently dead trees, and long-dead trees. The dynamic MOE of lumber from dead trees decreased by 6.6% in recently dead and 4.5% in long-dead trees compared to living trees, with no differences in wood stiffness observed between recently dead and long-dead trees. Similar results were reported by Barrette et al. [77]. These authors assessed the quality of lumber from dead black spruce trees based on the Hunter degradation classes: Hunter 1–2 for living or declining trees, Hunter 3 for recently dead trees, and Hunter 4 for dead trees with peeling bark. The results indicated a decrease in the bending MOR and MOE of 2 × 4 and 2 × 6 lumber from dead trees (Hunter 3 and 4) compared with lumber from living trees, with no significant difference between Hunter 3 and 4 [77]. Furthermore, Barrette et al. [78] observed a decrease in lumber grade with increasing Hunter class. Living trees (Hunter 1–2) yielded approximately 74% of lumber (after drying) that met the standards for No. 2 grade or better, according to the National Lumber Grades Authority [47]. Their results also showed that recently dead trees (Hunter 3) can still be economically profitable, yielding about 60% of lumber (dry) in the No. 2 grade or higher and a recovery volume comparable to that of living trees. In contrast, trees classified as Hunter 4 exhibited lower quality, yielding only 40% of lumber (dry) rated as No. 2 or better; therefore, their harvesting was not recommended [78].

Research on lumber from MPB outbreaks has focused on topics such as log processing and yield, mechanical properties of blue-stained wood, or strategies to mitigate low MC and blue stain in wood. A survey of sawmills in the Western United States found that cracks and checks are the most damaging defects affecting sawlog volume from MPB-killed trees, whereas blue stain has a minor impact. Sawlog volume can decrease from 85% in green stands to 36% in gray stands [67]. To address this issue, imaging techniques such as X-ray/CT scanners have been proposed to detect internal checks [79] and optimize log sorting and sawing processes. In fact, some modern sawmills in Canada and the United States already use this technology. The low MC of MPB-affected logs also raises major concerns for sawmills. It can cause breakage during handling and processing and increase the risk of fires and explosions in mills due to the high combustibility of dry sawdust [67]. Another study by Lum et al. [80] examined the mechanical properties of blue-stained sapwood. The results revealed that blue stain had little or no effect on the wood’s toughness and bending properties. Additionally, the study showed that the grip capacity of truss plate connectors improved, suggesting that this type of wood can be used in structural applications. Other research focused on strategies to minimize the impact of over-drying on lumber quality. Wood affected by MPB, which sometimes has an MC below 19%, must comply with phytosanitary regulations that require heat treatment at 56 °C for 30 min. Because standard kiln schedules are long, they can lead to excessive drying and reduced lumber quality. Cai and Oliveira [81] have proposed the use of low-pressure steam or cold-water spray log humidification systems, which offer advantages by reducing over-drying and improving lumber quality.

Blue-stained wood has also been evaluated for use in appearance-grade and painted products, such as siding. To increase the market acceptability and value of blue-stained wood from MPB-killed trees, extensive research has focused on improving its visual quality [82–85]. Studies have investigated methods to remove or reduce discoloration through chemical and photo-assisted bleaching, glow-discharge plasma treatment, heat treatment or finishing systems. For instance, Evans et al. [82] demonstrated that high-concentration sodium hypochlorite can significantly diminish blue staining in lodgepole pine lumber. However, the treated wood remained noticeably darker than unstained sapwood, indicating that a secondary brightening step is required to restore a more natural appearance. A combined treatment involving light exposure followed by sodium hypochlorite bleaching has shown improved stain removal without excessive darkening; however, this method is slow, costly, and challenging for industrial use [83]. Pre-plasma treatment followed by sodium hypochlorite bleaching can also remove nearly all visible blue stains, provided that practical atmospheric plasma systems are developed to increase efficacy [84]. Cai and Cai [85] proposed using heat treatments to improve the dimensional stability and visual appeal of blue-stained wood by darkening it, thereby masking blue stains; however, these treatments may negatively impact mechanical strength. In British Columbia, blue-stained lodgepole pine has even been marketed as a specialty product known as “denim wood”, highlighting the potential niche markets for aesthetically unique materials derived from disturbance-affected forests. A prominent example of MPB-affected wood utilization is the Richmond Olympic Oval, constructed for the 2010 Olympic Winter Games in British Columbia. This facility has one of the largest wooden roofs in the world, built from a combination of hybrid glulam, steel arches, and wave panels made from MPB-affected wood [86]. It is important to note, however, that blue stain increases wood permeability (water uptake), which can negatively affect the performance of surface treatments such as paints and finishes and elevate the risk of biological deterioration during outdoor use.

### Engineered Wood Products

#### Cross-Laminated Timber

CLT is a multi-layered wood panel composed of softwood lumber blocks bonded side-by-side with a structural adhesive. Typically, these panels consist of three, five, or more layers arranged in odd numbers, with the layers oriented perpendicularly to each other, and the outer layers aligned parallel [87]. This orthogonal configuration improves mechanical properties and enables the use of low-quality wood. By minimizing the negative effects of cracks and rot, which are commonly found in damaged or dead trees, CLT becomes a valuable product for maximizing the value of wood from natural disturbances [27]. Wood damaged by insects, such as MPB, can be acceptable for use in the core layers, where appearance is not a concern [88, 89]. A concern is the increased permeability of blue-stained wood, particularly in MPB-attacked wood where staining is severe, which could increase moisture absorption during service, increasing the likelihood of decay. According to Heo [90], engineered wood products are effective solutions for adding value to wood from MPB-killed trees, though they require adjustments to manufacturing parameters.

Ma et al. [27] demonstrated that CLT panels made from SBW-attacked trees can meet the performance requirements outlined in the ANSI/APA PRG 320–2019 standard [91], with only minimal impact on bending properties. These authors used white spruce wood from various tree categories (living, recently dead, or long dead) to produce three-layer CLT panels and to evaluate their bending and shear properties. Although the bending properties of panels were slightly decreased in dead tree categories, they remained statistically equal to those made from living trees. Moreover, all panels exceed the PRG 320 standard for Grade E3 (commonly used in non-critical structural applications) for MOE and met the Grade E1 (high-performance structural applications) for MOR. The shear modulus was more affected by the tree condition, with reductions of about 22% in the recently dead trees and 17% in the long-dead trees. However, even with these reductions, panels exceed the PRG 320 grade E1 reference.

#### Laminated Veneer Lumber and Plywood

The key factors limiting the use of damaged or dead trees for peeling or slicing into veneers are low MC and deep surface checks, which negatively affect veneer yields and full-sheet recovery [92, 93]. Several studies conducted by Wang and co-authors explored the use of wood from trees damaged or killed by the MPB to produce LVL and plywood [93–97]. Some issues with the use of such wood in LVL include high WA and thickness swelling (TS) of panels, as well as the presence of blue stain in veneers [94]. To address these issues, Wang and Chui [94, 95] investigated the properties of LVLs produced with MPB-affected lodgepole pine veneer that was impregnated with a low-solid-content PF resin. They evaluated two veneer grades: high-grade (E1) and low-grade (E2). Their results showed that brown-colored PF resin impregnation of the outer-layer veneer improved dimensional stability, increased hardness, shear strength, and MOR, and improved the overall appearance of LVLs, masking blue stain. MOE improvements were achieved by using a one-layer impregnated veneer for grade E1 and a double-layer impregnated veneer for grade E2 on the face and back sides.

In plywood production, previous research has primarily focused on process parameters such as log peeling, veneer sorting and drying, and panel manufacturing [93, 96, 97]. Despite the low MC of veneers from MPB-attacked wood, these veneers exhibit less moisture variability, enabling more efficient sorting and decreasing drying time by 25–35%. However, low MC and cracks can cause veneer ribbon breakage during peeling, and blue stain decrease veneer recovery and its visual grade [96]. In terms of plywood performance, Wang et al. [93] reported that plywood made from MPB-attacked lodgepole pine displayed enhanced glueability and higher MOE/MOR compared to that from spruce-pine-fir (SPF) veneers. Furthermore, Wang [97] investigated the viability of utilizing veneer from MPB-affected lodgepole pine in specialty plywood applications (e.g., concrete forms, industrial decks, and container floors). Their results indicated that plywood made entirely from MPB-affected wood exhibited better glueability than plywood made from Douglas-fir, but it also exhibited higher TS and WA. Ultimately, their study concluded that a mixed composition, incorporating Douglas-fir veneers for the outer layers and MPB veneers for the inner layer, significantly improved the plywood’s dimensional stability, aesthetic appeal, and overall performance.

### Wood-based Panels

#### Oriented Strand Board

Research on fire-damaged wood for OSB production is limited, with few studies conducted by Moya et al. [16]. They used fire-damaged jack pine wood to produce single-layer strand boards bonded with phenol-formaldehyde (PF) resin. Trees aged 30–37 years were harvested within 3 weeks after the fire, debarked, and the charred portion removed. The study evaluated the effect of four burn patterns (unburnt, low, moderate, and severe outer-bark damage) on panel properties. The level of fire damage did not significantly affect panel performance, and all panels met the mechanical properties outlined in CSA O437. However, chemical analysis of wood showed increased Klason lignin and decreased glucose content (expressed as weight%) in severely damaged trees. These changes may explain trends toward improved TS and WA and reduced MOR and MOE. This is likely due to decreased hemicellulose content, the most hydrophilic polymer in wood cells. Moya et al. [16] also evaluated the effect of using fire-damaged red pine (*Pinus resinosa*) from various fire intensities and at various proportions of charred bark on the properties of strand boards bonded with polymeric diphenylmethane diisocyanate (pMDI). Trees were harvested within 2 months following an intense forest fire. The incorporation of up to 10% of bark, regardless of fire severity, did not negatively affect the bending properties of the panels. However, exceeding this threshold led to a decrease in MOE and MOR. Additionally, both bending properties and IB strength met CSA requirements, regardless of the level of fire damage. An increase in bark content decreased the TS and WA of panels. However, most panels exceeded the CSA standard’s maximum permitted TS value. The bark-free strand boards made from fire-damaged red pine and bonded with pMDI demonstrated better TS performance than those made from fire-damaged jack pine bonded with PF. These results indicate that fire-damaged wood from both pine species is suitable for OSB manufacturing; however, selecting the right adhesive is essential to ensure that the panels comply with current TS standards.

Extensive MPB outbreaks in Western Canada over the past few decades have prompted several studies investigating the potential of MPB-affected wood for producing OSB. OSBs are traditionally made from southern yellow pine and trembling aspen, but lodgepole pine can also be used [90]. The main challenges associated with using MPB-affected wood for OSB, mainly in the gray stage, are the low MC, greater brittleness, increased cracking, and increased permeability, which contribute to excessive particle breakage during flaking. These characteristics adversely impact strand quality, resulting in a higher proportion of fines. An increased proportion of fines adversely affects OSB properties, increases adhesive consumption, and elevates production costs [31, 36]. To address these challenges, Feng and Knudson [31] investigated the effects of rehydrating lodgepole pine logs from standing dead trees that had been dead for 20 years on the quality of OSB strands. Their research demonstrated that spraying the logs with water for 10 days resulted in an MC, particularly in the sapwood, comparable to that of freshly cut lodgepole pine. This treatment resulted in a 28% reduction in fine production and a 45% increase in large strand production. Another concern with wood from MPB outbreaks is its elevated permeability, which facilitates WA and increases TS in OSB panels. Previous research shows that OSB made entirely of MPB-affected wood from standing dead trees that had been dead for 2 to 20 years exhibits low dimensional stability [69]. Semple et al. [36] evaluated various water-repellent surface treatments to improve the dimensional stability of industrial OSB panels made from 65% MPB-affected wood and 35% sound aspen wood. Commercial wax emulsions and linseed oil-based coatings mixed with hydrophobic waxes (e.g., carnauba, paraffin) or expandable plastic microspheres, provided short-term water resistance to OSB panels; however, panel performance declined after long-term water exposure. Additionally, blue stain, although it does not affect wood strength, may alter surface chemistry [53], thereby interfering with strand adhesion, wettability, and overall panel performance. Early studies emphasize the importance of developing solutions to address the low MC of MPB-affected wood for panel production. Additionally, further research is needed to better understand the effect of blue stain on panel adhesion and properties. It is also essential to assess how the changing properties of MPB-affected wood over time impact the production of wood-based panels [69]. However, since that time, there has been little research addressing these issues.

#### Particleboard

Research on the use of wood from dead trees in particleboard production began in the 1980s. According to Lowery [92], both dead lodgepole pine and western white pine trees are acceptable materials for this purpose. Particleboards made from wood from dead trees can have properties comparable to those produced from green, sound wood and can bond effectively with urea-formaldehyde (UF) or PF resins; however, they exhibit a high linear expansion. Moreover, wood from dead trees is harder and more brittle, requiring about 30% more energy to process into particles than green, sound wood. On the positive side, the lower MC of wood from dead trees can reduce drying energy and drying costs. Additionally, wood waste from dead trees can be used as fuel for mills, helping offset energy costs [92]. Bekhta et al. [98] investigated the impact of dead-tree wood particles, composed of 75% softwood and 25% hardwood, on particleboard production. The study evaluated how different proportions of dead-tree wood particles (25%, 50%, 75%, and 100%) and resin type (UF or UF + melamine-urea-formaldehyde – MUF) affect the physical and mechanical properties and formaldehyde emissions of panels. The MOR and MOE of panels made entirely from dead-tree wood particles decreased by approximately 27% and 23%, respectively. Additionally, the IB strength decreased significantly from 26% to 74% as the proportion of dead-tree wood particles increased from 25% to 100%. TS and WA increased by 21% and 11%, respectively, in panels made from 100% dead-tree wood particles, compared with control panels. However, panels containing up to 25% dead-tree wood particles exhibited no significant loss in properties when bonded with UF resin. Furthermore, the addition of 3% MUF improved both the mechanical and physical properties of panels made entirely from dead-tree wood particles. The notable advantage of using dead-tree wood in particleboards was the reduction in formaldehyde emissions. It was attributed to the low MC and low extractive content of the dead-tree wood, as well as its long storage time, which helps reduce volatile organic compound emissions. The emissions decreased by about 35% in particleboards made from 100% dead-tree wood particles bonded with UF resin, and by about 39% with the addition of 3% MUF resin. The study did not consider variables such as tree species, age, degradation stage, or time since the tree’s death, which could impact the performance of the resulting particleboards. However, these results provide helpful insights into the use of dead-tree wood in particleboard manufacturing.

#### Fiberboard

Akgül et al. [17] investigated the use of fire-damaged Scots pine wood in MDF production, testing various ratios of burned wood from 0% to 100% mixed with unburned wood (50% beech and 50% oak). The study showed that fire-damaged fibers did not adversely affect the bending properties or the IB strength of the panels. In contrast, fire-damaged fibers decreased the dimensional stability of the panels with increases in TS from 77% to 143%, depending on the fire-damaged fiber proportion. Furthermore, the fire-damaged fibers affected the finishing quality of the panels, resulting in increased surface roughness and darker coloration. The study found that up to 50% of fire-damaged pine fibers can be used in MDF production without compromising mechanical properties. The performance of panels in terms of TS can be improved by adjusting the resin content or pressing conditions.

According to Byrne et al. [69], the low MC of the wood affected by MPB can be a concern for OSB manufacturing; however, it may offer advantages for MDF manufacturing. The drier and lighter material could reduce transportation and production costs, particularly by reducing drying time. Vititnev and Kazitsin [99] investigated the production of binderless fiberboard using wood from Siberian fir (*Abies sibirica*) trees affected by the bark beetle *Polygraphus proximus*, a major pest in Siberian and central European Russia. Their study focused on trees that had been dead for up to 19 years. The wood fibers were obtained using a thermomechanical pulping method and refined with a single-disc refiner. The results indicated that the degree of fiber refining increased with the time since the tree’s death, while the specific power consumption gradually decreased to 70%. Notably, wood from trees dead between 5 and 12 years produced high-quality fibers, exhibited an improved slenderness ratio, and had a higher proportion of medium fiber fractions compared to fibers from living trees. Fiberboard produced from trees that had been dead for 5 to 12 years demonstrated improved bending properties and IB strength compared to that made from living trees’ fibers. In contrast, fibers from trees that had been dead for 19 years resulted in fiberboards with reduced TS (probably due to a decreased hemicellulose content resulting from wood degradation), along with surface defects such as dark spots. Overall, these results indicate that dead-tree wood requires less energy to refine because its fibers become more susceptible to mechanical breakdown and are weakened by biological degradation over time. A similar trend was reported by Koran and Nlombi [100], who found a 15% reduction in refining energy during chemi-thermomechanical pulping of balsam fir wood from trees that had been dead for 3 years following an SBW attack.

## Conclusions

In recent years, forest fires have intensified and become more frequent, especially in temperate and boreal forests, while unprecedented SBW outbreaks have been reported in Eastern Canada. Although MPB outbreaks have declined since 2019, they remain cyclical, indicating potential future resurgences. From 2023 to 2025, these disturbances have affected over 60 million hectares of forest area in Canada, causing significant tree damage and mortality.

The quality of disturbance-affected wood decreases following tree mortality and declines further over time due to ongoing degradation. Forest fires immediately reduce wood quality, while SBW and MPB lead to gradual deterioration before tree death occurs. High-quality wood can typically be recovered within the first year after a fire or MPB infestation. In contrast, for the SBW, the timing of harvesting may be delayed because trees die after several years of intense defoliation. The heterogeneous levels of damage in a stand following forest fires and insect infestations are a major challenge for wood recovery and its use for the manufacture of various products.

Wood from dead trees exhibits lower and variable MC and more defects than sound wood, challenging sawmill processing and product manufacturing. Wood defects, such as insect holes, blue stains, checks, and decay, significantly impact lumber recovery and grades. However, this type of wood can still be used in high-value products such as CLT panels. Low MC and checks are the major issues in log conversion into veneers for LVL or plywood production, affecting veneer yields and full-sheet recovery.

Forest fires impact certain wood properties and pose major problems for sawmills, especially regarding low MC and the formation of char and soot. There is limited research on post-fire wood quality over time and its processing for various products. Additionally, the relationship between tree species, size, and age and the quality of fire-damaged wood across different wood products has not been thoroughly investigated.

Using wood from MPB outbreaks to manufacture OSB presents challenges related to strand production and panel properties. The low MC of the logs results in a high proportion of fines during flaking when log humidification is inadequate. Furthermore, the wood’s high permeability, caused by blue-staining fungi, results in panels that are less water-resistant and less dimensionally stable. Similar issues are expected for wood from trees affected by forest fires and SBW infestations.

Although the suitability of disturbance-affected wood for products like lumber, LVL, plywood, or OSB decreases over time, it can ultimately be transformed into value-added products such as particleboard or fiberboard. These panels can perform well with a limited amount of disturbance-affected wood, even when sourced from trees that have been dead for several years. A key benefit of using dead trees in particleboard production is their significantly lower formaldehyde emissions, which is advantageous for the panel manufacturing industry. However, improvements in the dimensional stability of panels are needed. For fiberboard production, dead trees can provide high-quality fiber and require less specific energy in the refining process. Yet very few studies have explored the use of dead trees in the manufacture of these panels.

To fully realize the potential of disturbance-affected wood, future research should focus on how its properties change over time and how these changes impact the production of various products. Additionally, studies should aim to optimize log sorting and conversion processes and to adapt manufacturing techniques to account for the unique characteristics of disturbance-affected wood. This will help ensure the performance and quality of the final products.

## Key References

- Cunningham CX, Williamson GJ, Bowman DMJS. Increasing frequency and intensity of the most extreme wildfires on Earth. Nat Ecol Evol. 2024;8:1420–1425. https://doi.org/10.1038/s41559-024-02452-2.
  - ◦ **This paper provides solid insights into the frequency and intensity of wildfires worldwide**,** showing a rapid increase in their occurrence in boreal and temperate conifer forests.**
- Boulanger Y, Arseneault D, Bélisle AC, Bergeron Y, Boucher J, Boucher Y, et al. La saison des feux de forêt 2023 au Québec: un aperçu des conditions extrêmes, des impacts, des leçons apprises et des considérations pour l’avenir. Can J For Res. 2025;55:1–23. https://doi-org.acces.bibl.ulaval.ca/10.1139/cjfr-2024-0230.
  - ◦ **A comprehensive publication on the economic**,** environmental**,** and social impacts of the 2023 fire season in Eastern Canada. It examines the effects of these fires on the forest sector**,** fire management**,** wildlife (specifically the boreal caribou)**,** and First Nations communities.**
- Marttila J, Möttönen V, Haapala A, Ylimäki P, Kilpeläinen P, Verkasalo E. Wood material properties of forest fire-damaged Norway spruce and Scots pine for mechanical wood processing in Finland. Appl Sci. 2024;14:238. https://doi.org/10.3390/app14010238.
  - ◦ **Recent and comprehensive research on the use of wood from forest fires. It provides insights into the properties of fire-damaged wood and the challenges related to its processing.**
- Ma Y, Wang X, Begel M, Dai Q, Dickinson Y, Xie X, et al. Flexural and shear performance of CLT panels made from salvaged beetle-killed white spruce. Constr Build Mater. 2021;302:124381. https://doi.org/10.1016/j.conbuildmat.2021.124381.
  - ◦ **This publication is one of the few resources released in recent years addressing the use of wood from both living and dead trees following spruce budworm infestations for wood products. It presents valuable insights into the bending properties of lumber and cross-laminated timber panels**,** as well as their compliance with current standards.**
- Houndode DJ, Krause C, Morin H. Predicting balsam fir mortality in boreal stands affected by spruce budworm. For Ecol Manag. 2021;496:119408. https://doi.org/10.1016/j.foreco.2021.119408.
  - ◦ **A recent study examining how defoliation by spruce budworm affects balsam fir mortality. It considers factors such as defoliation duration and severity**,** forest composition**,** and stand age.**
- Bekhta P, Kozak R, Gryc V, Sebera V, Tippner J. Effects of wood particles from deadwood on the properties and formaldehyde emission of particleboards. Polymers. 2022;14(17):3535. https://doi.org/10.3390/polym14173535.
  - ◦ **This is the only recent paper in the literature that evaluates the impact of using wood from dead trees in the manufacture of particleboard. It evaluates the physical and mechanical properties and formaldehyde emissions of panels produced from various proportions of wood particles from dead trees.**
- Vititnev A, Kazitsin S. Using Siberian fir (*Abies sibirica*) dead wood in wood fiberboard production. BioResources. 2025;20(3):5315–30.
  - ◦ **A recent study on the refining and fiber quality of dead trees after bark beetle infestation**,** along with the physical and mechanical properties of fiberboard produced from the wood of these trees.**

## Data Availability

No datasets were generated or analysed during the current study.

## References

[1] FAO. Global Forest Resources Assessment 2025. Rome: FAO; 2025. 10.4060/cd6709en.

[2] Jones MW, Kelley DI, Burton CA, Di Giuseppe F, Barbosa MLF, Brambleby E, et al. State of wildfires 2023–2024. Earth Syst Sci Data. 2024;16:3601–85. 10.5194/essd-16-3601-2024.

[3] Zheng B, Ciais P, Chevallier F, Chuvieco E, Chen Y, Yang H. Increasing forest fire emissions despite the decline in global burned area. Sci Adv. 2021;7:eabh2646. 10.1126/sciadv.abh2646. https://www.science.org/doi/. 34559570 10.1126/sciadv.abh2646 PMC8462883

[4] Cunningham CX, Williamson GJ, Bowman DMJS. Increasing frequency and intensity of the most extreme wildfires on Earth. Nat Ecol Evol. 2024;8:1420–5. 10.1038/s41559-024-02452-2. 38914710 10.1038/s41559-024-02452-2

[5] Copernicus Atmosphere Monitoring Service. CAMS Global wildfires review 2024: a harsh year for the Americas [Internet]. Brussels: European Union. 2024 [cited 2025 June 30]. Available from: https://atmosphere.copernicus.eu/cams-global-wildfires-review-2024-harsh-year-americas

[6] Our World in Data. Wildfires [Internet]. Global Change Data Lab; 2024 [cited 2025 Octobre 30]. Available from: https://ourworldindata.org/wildfires

[7] Boulanger Y, Arseneault D, Bélisle AC, Bergeron Y, Boucher J, Boucher Y, et al. La saison des feux de forêt 2023 au Québec: un aperçu des conditions extrêmes, des impacts, des leçons apprises et des considérations pour l’avenir. Can J Res. 2025;55:1–23. 10.1139/cjfr-2024-0230. https://doi-org.acces.bibl.ulaval.ca/.

[8] World Resources Institute. Global Forest Watch – Dashboard: Fires [Internet], Washington DC. World Resources Institute; 2025 [cited 2025 October 21]. Available from: https://www.globalforestwatch.org/dashboards/global/

[9] Canadian Interagency Forest Fire Centre. CIFFC homepage [Internet]. Winnipeg (MB): CIFFC; 2025 [cited 2025 October 21]. Available from: https://ciffc.net/

[10] Radio-Canada. Feux historiques: le Forestier en chef exige une baisse de la récolte de bois [Internet]. Montréal: Société Radio-Canada; 2023 [cited 2025 July 04]. Available from: https://ici.radio-canada.ca/nouvelle/2030985/impacts-feux-forets-possibilite-forestiere

[11] Bureau du Forestier en chef. Possibilités forestières 2023–2028: recommandation d’une mise à jour à la suite des feux de forêt 2023. Roberval (QC). Bureau du Forestier en chef; 2023. p. 8.

[12] Dussault L, Tremblay M. Récolte du bois brûlé dans le Nord-du-Québec « C’est vraiment un marathon » [Internet]. Montréal: La Presse; 2023 [cited 2025 October 21]. Available from: https://www.lapresse.ca/actualites/regional/la-presse-en-abitibi/la-course-au-bois-brule/2023-08-21/recolte-du-bois-brule-dans-le-nord-du-quebec/c-est-vraiment-un-marathon.php

[13] Barrette J, Thiffault E, Saint-Pierre F, Wetzel S, Duchesne I, Krigstin S. Dynamics of dead tree degradation and shelf-life following natural disturbances: can salvaged trees from boreal forests ‘fuel’ the forestry and bioenergy. sectors? Forestry. 2015;88:275–90. 10.1093/forestry/cpv007.

[14] Mansuy N, Barrette J, Laganière J, Mabee W, Paré D, Gautan S, et al. Salvage harvesting for bioenergy in Canada: From sustainable and integrated supply chain to climate change mitigation. WIREs Energy Environ. 2018;7:e298. 10.1002/wene.298.

[15] Marttila J, Möttönen V, Haapala A, Ylimäki P, Kilpeläinen P, Verkasalo E. Wood material properties of forest fire-damaged Norway spruce and Scots pine for mechanical wood processing in Finland. Appl Sci. 2024;14:238. 10.3390/app14010238.

[16] Moya L, Winandy JE, Tze WTY, Ramaswamy S. Use of fire-impacted trees for oriented strandboards. Prod J. 2008;58(6):45–52.

[17] Akgül M, Ayrilmis N, Çamlibel O, Korkut S. Potential utilization of burned wood in manufacture of medium density fiberboard. J Mater Cycles Waste Manag. 2013;15:195–201. 10.1007/s10163-012-0108-3.

[18] Puettmann ME. Carbon analysis of wood composite panels. For Prod J. 2022;72(2):112–5. 10.13073/FPJ-D-22-00010.

[19] Kazulis V, Muizniece I, Zihare L, Blumberga D. Carbon storage in wood products. Energy Procedia. 2017;128:558–63. 10.1016/j.egypro.2017.09.009.

[20] Faluyi MO, Irmak S. Northeastern American forests: natural disturbances, climate change impact, and the utilization of increasingly damaged forest trees for biofuel production. Forests. 2023;14:2409. 10.3390/f14122409.

[21] Nappi A, Déry S, Bujold F, Chabot M, Dumont M-C, Duval J. La récolte dans les forêts brûlées – Enjeux et orientations pour un aménagement écosystémique. Québec: Ministère des Ressources naturelles et de la Faune, Direction de l’environnement et de la protection des forêts; 2011. p. 51.

[22] Natural Resources Canada. The State of Canada’s Forests: Annual Report 2024. Ottawa: Canadian Forest Service. 2024. [cited 2025 November 11]. Available from: https://natural-resources.canada.ca/our-natural-resources/forests/state-canadas-forests-report/16496

[23] Lemay A, Barrette J, Krause C. Balsam Fir (*Abies balsamea* (L.) Mill.) Wood Quality after Defoliation by Spruce Budworm (*Choristoneura fumiferana* Clem.) in the Boreal Forest of Quebec, Canada. Forests. 2022;13:1926. 10.3390/f13111926.

[24] Natural Resources Canada. Spruce budworm [Internet]. Ottawa (ON): Government of Canada. 2025a [cited 2025 September 05]. Available from: https://natural-resources.canada.ca/forest-forestry/insects-disturbances/spruce-budworm

[25] Natural Resources Canada. Mountain pine beetle [Internet]. Ottawa (ON): Government of Canada. 2025b [cited 2025 October 28]. Available from: https://natural-resources.canada.ca/forest-forestry/insects-disturbances/mountain-pine-beetle

[26] Paixao C, Krause C, Morin H, Achim A. Wood quality of black spruce and balsam fir trees defoliated by spruce budworm: A case study in the boreal forest of Quebec, Canada. Ecol Manage. 2019;437:201–10. 10.1016/j.foreco.2019.01.032.

[27] Ma Y, Wang X, Begel M, Dai Q, Dickinson Y, Xie X, et al. Flexural and shear performance of CLT panels made from salvaged beetle-killed white spruce. Constr Build Mater. 2021;302:124381. 10.1016/j.conbuildmat.2021.124381.

[28] Mushakhian S, Ouhimmou M, Rönnqvist M. Salvage harvest planning for spruce budworm outbreak using multistage stochastic programming. Can J Res. 2020;50(10):953–65. 10.1139/cjfr-2019-0283. https://doi-org.acces.bibl.ulaval.ca/.

[29] Ministère des Ressources naturelles et des Forêts (MRNF). Insectes, maladies et feux dans les forêts du Québec en 2024. Québec: Direction de la protection des forêts. 2025a. Report No.: ISBN 978-2-555-00861-8. Available from: https://www.quebec.ca/agriculture-environnement-et-ressources-naturelles/forets/protection-forets/donnees-feux-insectes-maladies

[30] Kim J-J, Allen EA, Humble LM, Breuil C. Ophiostomatoid and basidiomycetous fungi associated with green, red, and grey lodgepole pines after mountain pine beetle (*Dendroctonus ponderosae*) infestation. Can J Res. 2005;35(2):274–84. 10.1139/x04-178.

[31] Feng MW, Knudson RM. Effect of log rehydration on quality of OSB strands manufactured from beetle-killed lodgepole pine. Prod J. 2007;57(1–2):35–42.

[32] Fettig CJ, Hood SM, Runyon JB, Stalling CM. Bark beetle and fire interactions in western coniferous forests: research findings. Fire Mang Today. 2021;70(1):14–23.

[33] Province of British Columbia. 1999–2015 mountain pine beetle outbreak [Internet]. British Columbia (CA): Province of British Columbia; 2025 [cited 2025 December 13]. Available from: https://www2.gov.bc.ca/gov/content/industry/forestry/managing-our-forest-resources/forest-health/forest-pests/bark-beetles/mountain-pine-beetle

[34] Montana State University. Mountain pine beetle [Internet]. Bozeman (MT): Montana State University; 2025 [cited 2025 November 18]. Available from: https://www.montana.edu/extension/Full_HTML_Pubs/a-guide-to-pests-problems-and-identification-of-ornamental-shrubs-and-trees-in-montana/insects/mountain-pine-beetle.html

[35] Bogdanski B, Sun L, Peter B, Stennes B. Markets for forest products following a large disturbance: opportunities and challenges from the mountain pine beetle outbreak in western Canada. Victoria (BC): Natural Resources Canada, Canadian Forest Service, Pacific Forest Centre; 2011. Inf. Rep. BC-X-429.

[36] Semple K, Cullis I, Evans P. Improving the stability of oriented strand board manufactured from mountain pine beetle wood. Victoria (BC): Natural Resources Canada, Canadian Forest Service, Pacific Forestry Centre; 2009. Mountain Pine Beetle Working Paper 2009-18. 61 p. Available from: https://ostrnrcan-dostrncan.canada.ca/entities/publication/0b3160a0-6afa-4515-8c66-9f4c5d06b479

[37] Keeley JE. Fire intensity, fire severity and burn severity: a brief review and suggested usage. Int J Wildland Fire. 2009;18(10):116–26.

[38] Ministère des Ressources naturelles et des Forêts (MRNF). Information sur les plans d’aménagement spéciaux et l’aide financière à l’intention des organismes désignés. Québec: Gouvernement du Québec. 2025b. 52 p. French. ISBN 978-2-550-92615-3.

[39] Barrette J, Fortier S. Évolution des dommages causés par le longicorne et les champignons de dégradation sur les produits du bois à la suite d’un feu de printemps. Québec: Gouvernement du Québec, ministère des Ressources naturelles et des Forêts, Direction de la recherche forestière; 2023. Avis technique SSRF-32. 12 p.

[40] Forest Products Laboratory. Wood handbook: wood as an engineering material. General Technical Report FPL-GTR-190. Madison (WI): U.S. Department of Agriculture, forest Service, forest Products Laboratory. 2010. 508 p.

[41] Saint-Germain M, Greene DF. Salvage logging in the boreal and cordilleran forests of Canada: Integrating industrial and ecological concerns in management plans. Chron. 2009;85(1):120–34. 10.5558/tfc85120-1. https://doi-org.acces.bibl.ulaval.ca/.

[42] Costello SL, Jacobi WR, Negron JF. Emergence of Buprestidae, Cerambycidae, and Scolytinae (Coleoptera) from mountain pine beetle-killed and fire-killed ponderosa pines in the Black Hills, South Dakota, USA. Coleopt Bull. 2013;67(2):149154. 10.1649/0010-065X-67.2.149.

[43] Siegert C, Clay N, Pace K, Vissa S, Hofstetter RW, Leverón O, et al. Bark beetle-driven community and biogeochemical impacts in forest ecosystems: a review. Ann Entomol Soc Am. 2024;117(3):163–83. 10.1093/aesa/saae009.

[44] Bélanger S, Bauce É, Berthiaume R, Long B, Labrie J, Daigle L-F, et al. Effect of temperature and tree species on damage progression caused by whitespotted sawyer (Coleoptera: Cerambycidae) larvae in recently burned logs. J Econ Entomol. 2013;106(3):1331–8. 10.1603/EC12372. 23865199 10.1603/ec12372

[45] Natural Resources Canada. White-spotted sawyer beetle (*Monochamus scutellatus*). Canadian Forest Service – Tree Insect and Disease Fact Sheets [Internet]. Ottawa (ON): Natural Resources Canada; 2011 [cited 2025 November 11]. Available from: https://tidcf.nrcan.gc.ca/en/insects/factsheet/900

[46] Gervais DJ, Greene DF, Work TT. Causes of variation in wood-boring beetle damage in fire-killed black spruce (*Picea mariana*) forests in the central boreal forest of Quebec. Ecoscience. 2012;19(4):398–403. 10.2980/19-4-3568.

[47] National Lumber Grades Authority. Standard grading rules for Canadian lumber. Ottawa (ON): NLGA; 2022. p. 322.

[48] Tuo B, Lin L, van Rantwijk RS, van Logtestijn RSP, Goudzwaard L, Scheffers K, et al. Positive feedback from woodpeckers on deadwood decomposition via invertebrates. Curr Biol. 2025;35:2732–9. 40345194 10.1016/j.cub.2025.04.041

[49] Tingley MW, Montgomery GA, Wilkerson RL, Cluck DR, Sawyer SC, Siegel RB. Multi-trophic occupancy modeling connects temporal dynamics of woodpeckers and beetle sign following fire. PLoS One. 2023;18(3):e0281687. 10.1371/journal.pone.0281687. 36877704 10.1371/journal.pone.0281687 PMC9987826

[50] Nappi A, Drapeau P, Giroux J-F, Savard J-P. Sang use by foraging black-backed woodpeckers (*Picoides articus*) in a recently burned Eastern Boreal Forest. Auk. 2003;120(2):505–11.

[51] Broda M. Natural compounds for wood protection against fungi—a review. Molecules. 2020;25:3538. 10.3390/molecules25153538. 32748877 10.3390/molecules25153538 PMC7435604

[52] Goodell B, Nielsen G. Wood Biodegradation. In: Niemz P, Teischinger A, Sandberg D, editors. Springer handbook of wood science and technology. Cham: Springer Nature Switzerland AG; 2023. pp. 139–77.

[53] Kržišnik D, Lesar B, Thaler N, Humar M. Performance of bark beetle damaged Norway spruce wood against water and fungal decay. BioResources. 2018;13(2):3473–86.

[54] Goodell B, Qian Y, Jellison J. Fungal decay of wood: soft rot – brown rot – white rot. In: Schultz TP, Nicholas DD, editors. Development of commercial wood preservatives. ACS Symposium Series. Washington (DC): American Chemical Society; 2008. pp. 9–31.

[55] Mușat EC. Impact of forest fires on the trees and wood quality—a case study for a beech stand. Fire. 2024;7(9):325. 10.3390/fire7090325.

[56] Leverkus AB, Gustafsson L, Lindenmayer DB, Castro J, Benayas JMR, Ranius T, Simon Thorn S. Salvage logging effects on regulating ecosystem services and fuel loads. Front Ecol Environ. 2020;18(7):391–400. 10.1002/fee.2219.

[57] Houndode DJ, Krause C, Morin H. Predicting balsam fir mortality in boreal stands affected by spruce budworm. For Ecol Manage. 2021;496:119408. 10.1016/j.foreco.2021.119408.

[58] Bergeron Y, Leduc A, Joyal C, Morin H. Balsam fir mortality following the last spruce budworm outbreak in northwestern Quebec. Can J Res. 1995;25(8):1375–84. 10.1139/x95-150.

[59] Chabot M, Beaupré P, Fournier C, Roy M-E, Vaillancourt M-A, Jetté J-P, et al. L’aménagement forestier dans un contexte d’épidémie de la tordeuse des bourgeons de l’épinette – guide de référence pour moduler les activités d’aménagement dans les forêts privées. Québec: ministère des Forêts, de la Faune et des Parcs, Direction de l’aménagement et de l’environnement forestiers. Direction de la protection des forêts; 2015. p. 87.

[60] Ip DW, Pines IL, Westwood AR. Wood moisture content variation in white spruce defoliated by spruce budworm. Chron. 1996;72(2):176–80. 10.5558/tfc72176-2.

[61] Slippers B, Coutinho TA, Wingfield BD, Wingfield MJ. A review of the genus *Amylostereum* and its association with woodwasps. S Afr J Sci. 2003;99(1–2):70–4.

[62] Lewis K, Thompson D. Degradation of wood in standing lodgepole pine killed by mountain pine beetle. Wood Fiber Sci. 2011;43(2):130–42.

[63] Lewis KJ, Hartley I. Rate of deterioration, degrade and fall of trees killed by mountain pine beetle: a synthesis of the literature and experiential knowledge. Victoria (BC): Natural Resources Canada; 2005.

[64] Woo KL, Watson P, Mansfield SD. The effects of mountain pine beetle attack on lodgepole pine wood morphology and chemistry: implications for wood and fiber quality. Wood Fiber Sci. 2005;37(1):112–26.

[65] McFarling S, Byrne T. Characterising the dimensional stability, checking, and permeability of wood containing beetle-transmitted bluestain. Vancouver (BC): Forintek Canada Corp.; 2003. Report No.: R2003-0133.

[66] Cai L, Oliveira LC. Impact of mountain pine beetle (MPB) attack on drying characteristics of wood. Wood Fiber Sci. 2008;40(3):392–6.

[67] Loeffler D, Anderson N. Impacts of the mountain pine beetle on sawmill operations, costs, and product values in Montana. Prod J. 2018;68(1):15–24. 10.13073/FPJ-D-17-00041.

[68] Breuil C. Decay fungi and associated rates of decay in standing trees killed by mountain pine beetle. Victoria (BC): Natural Resources Canada, Canadian Forest Service, Pacific Forestry Centre; 2008. 25 p. (Mountain Pine Beetle Initiative working paper; 2008-11).

[69] Byrne T, Stonestreet C, Peter B. Chapter 9: Characteristics and utilization of post-mountain pine beetle wood in solid wood products. In: Safranyik L, Wilson B, editors. The mountain pine beetle: a synthesis of biology, management, and impacts on lodgepole pine. Victoria (BC): Natural Resources Canada, Pacific Forestry Centre; 2006. pp. 233–53.

[70] Zziwa A, Mukasa J, Kizito S. Structural suitability of 10-year old *Pinus caribaea* timber with a forest fire history in farm buildings. Agri Eng Int CIGR J. 2020;22(2):49–58.

[71] Bortoletto Júnior G, Moreschi JC. Physical-mechanical properties and chemical composition of *Pinus taeda* mature wood following a forest fire. Bioresour Technol. 2003;87:231–8. 10.1016/S0960-8524(02)00242-0. 12507861 10.1016/s0960-8524(02)00242-0

[72] Carvalho A. Madeiras salvadas de fogos florestais. Alcobaca (Portugal): Departamento de Tecnologia dos Produtos Florestais, Estação Nacional de Tecnologia dos Produtos Agrários/INIA; 1986.

[73] Lukina AL, Lisyatnikov ML, Lukin ML, Vatin N, Roshchina SR. Strength properties of raw wood after a wildfire. Mag Civ Eng. 2023;199(3):11906.

[74] Kolström T, Kellomäki S. Tree survival in wildfires. Silva Fenn. 1993;27(4):277–81.

[75] Chu Y. Burnt wood: recovering wood fibre from wildfires [Internet]. Pointe-Claire (QC): FPInnovations; 2022 [cited 2025 December 11]. Available from: https://web.fpinnovations.ca/burnt-wood-recovering-wood-fibre-from-wildfires/

[76] Marschall JM, Guyette RP, Stambaugh MC, Stevenson AP. Fire damage effects on red oak timber product value. Ecol Manag. 2014;320:182–9. 10.1016/j.foreco.2014.03.006.

[77] Barrette J, Pothier D, Duchesne I. Lumber and wood chips properties of dead and sound black spruce trees grown in the boreal forest of Canada. Forestry. 2015;88(1):108–20. 10.1093/forestry/cpu033.

[78] Barrette J, Pothier D, Auty D, Achim A, Duchesne I, Gélinas N. Lumber recovery and value of dead and sound black spruce trees grown in the North Shore region of Quebec. Ann Sci. 2012;69(6):603–15. 10.1007/s13595-011-0178-8.

[79] Brdicko J. Feasibility of optimizing log sorting and lumber manufacturing processes by using X-ray scanning to characterize check severity in mountain pine beetle-affected logs. Vancouver (BC): FPInnovations-Forintek Division; 2009. Report No.: MPB Working Paper 2009-25.

[80] Lum C, Byrne T, Casilla R. Mechanical properties of lodgepole pine containing beetle-transmitted blue stain. Prod J. 2006;56(6):45–50.

[81] Cai L, Oliveira LC. Evaluating the use of humidification systems during heat treatment of MPB lumber. Dry Technol. 2011;29(7):729–34.

[82] Evans PD, Palmer G, Chowdhury M. Bleaching treatments for blue-stained lodgepole pine affected by the mountain pine beetle *Dendroctonus ponderosae*. Holz Roh Werkst. 2007;65:485–6. 10.1007/s00107-007-0177-5.

[83] Stirling R, Morris PI. Photo-assisted bleaching of blue stained wood. Vancouver (BC): FPInnovations – Forintek Division; 2008. 17 p. https://library.fpinnovations.ca/link/fpipub41382

[84] Jamali A, Evans PD. Plasma treatment and bleaching to remove blue-stain from lodgepole pine sapwood. Eur J Wood Prod. 2013;71:675–7. 10.1007/s00107-013-0717-0. https://doi-org.acces.bibl.ulaval.ca/.

[85] Cai J, Cai L. Effect of thermal modification on mechanical and swelling properties and color change of lumber killed by mountain pine beetle. BioRes. 2012;7(3):3488–99.

[86] Naturally Wood. Richmond Olympic Oval [Internet]. Vancouver (BC): Naturally Wood; 2025 [cited 2025 October 28]. Available online: https://www.naturallywood.com/projects/richmond-olympic-oval/

[87] Brandner R, Flatscher G, Ringhofer A, Schickhofer G, Thiel A. Cross laminated timber (CLT): overview and development. Eur J Wood Prod. 2016;74:331–51. 10.1007/s00107-015-0999-5.

[88] Natural Resources Canada. Cross-laminated timber [Internet]. Ottawa (ON): Natural Resources Canada; 2025c [cited 2025 October 28]. Available from: https://natural-resources.canada.ca/forest-forestry/forest-industry-trade/taxonomy-wood-products

[89] Burrows JH. Resilient timber construction in the era of climate change: lessons from the mountain pine beetle [Internet]. Chapel Hill (NC): University of North Carolina at Chapel Hill; 2014 [cited 2025 October 28]. Available online: 10.17615/8dn0-he51

[90] Heo J. Potential international market for mountain pine beetle killed trees. Vancouver (BC): University of British Columbia, Faculty of Forestry; 2013. p. 27.

[91] APA – The Engineered Wood Association. Standard for Performance-rated Cross Laminated Timber. AINSI/APA PRG 320–2019. Tacoma (WA): APA; 2020.

[92] Lowery DP. The dead softwood timber resource and its utilization in the West. Ogden (UT): United States Department Agric For Service Intermt For Range Exp Stn. 1982;General Technical Report Int–125:18p.

[93] Wang BJ, Dai C, Wharton S. Impact of mountain pine beetle-attacked lodgepole pine logs on plywood manufacturing. Wood Fiber Sci. 2008;40(3):412–26.

[94] Wang BJ, Chui YH. Manufacturing of LVL using cost-effective resin impregnation and layup technologies. Wood Sci Technol. 2012a;46:1043–59. 10.1007/s00226-012-0465-z.

[95] Wang BJ, Chui YH. Performance evaluation of phenol formaldehyde resin-impregnated veneers and laminated veneer lumber. Wood Fiber Sic. 2012b;44(1):5–13.

[96] Wang BJ, Dai C. Impact of mountain pine beetle-attacked lodgepole pine logs on veneer processing. Wood Fiber Sci. 2008;40(3):397–411.

[97] Wang BJ. Increasing value recovery of mountain pine beetle (MPB)-attacked lodgepole pine logs for specialty plywood products. For Prod J. 2009;59(1/2):35–42.

[98] Bekhta P, Kozak R, Gryc V, Sebera V, Tippner J. Effects of wood particles from deadwood on the properties and formaldehyde emission of particleboards. Polymers. 2022;14(17):3535. 10.3390/polym14173535. 36080610 10.3390/polym14173535 PMC9460321

[99] Vititnev A, Kazitsin S. Using Siberian fir (*Abies sibirica*) dead wood in wood fiberboard production. BioResources. 2025;20(3):5315–30.

[100] Koran Z, Nlombi K. Chemithermomechanical pulping characteristics of budworm-killed trees. Wood Fiber Sci. 1994;26(4):489–95.

